# Gene Therapy for Corneal Diseases

**DOI:** 10.3390/biotech15040080

**Published:** 2026-09-22

**Authors:** Basanta Bhujel, Kyu Sang Eah, Soon Suk Kang, Ho Seok Chung, Jae-Yong Kim

**Affiliations:** Department of Ophthalmology, University of Ulsan College of Medicine, Asan Medical Center, Seoul 05505, Republic of Korea; basantabhujel86@gmail.com (B.B.); kseah0124@gmail.com (K.S.E.); joy_sskang@naver.com (S.S.K.); chunghoseok@gmail.com (H.S.C.)

**Keywords:** corneal diseases, gene therapy, gene delivery, gene editing, corneal regeneration

## Abstract

Corneal diseases are a major cause of blindness and visual impairment worldwide. Although conventional pharmacological therapies can alleviate mild to moderate corneal disorders, they often fail to restore vision in advanced disease, leaving corneal transplantation as the current standard treatment. However, donor tissue shortages, graft rejection, and surgical complications continue to limit its long-term success, highlighting the urgent need for effective nonsurgical therapeutic strategies. Gene therapy has emerged as a promising approach for treating inherited and acquired corneal diseases by targeting the underlying molecular mechanisms that drive disease progression. Recent advances in genome-editing technologies, gene-delivery systems, and vector engineering have substantially expanded the clinical potential of gene-based interventions. This review summarizes current progress in gene-editing strategies, gene-delivery platforms, and their therapeutic applications across a broad spectrum of corneal diseases. In addition, we discuss the major challenges and future perspectives associated with delivery efficiency, safety, vector optimization, and clinical translation. Collectively, these advances highlight the potential of gene therapy to transform the management of corneal diseases by providing safe, targeted, and long-lasting therapeutic strategies for restoring corneal function and vision.

## 1. Introduction

The cornea is a transparent, avascular tissue that serves as the principal refractive component of the eye, contributing approximately two-thirds of its total refractive power while providing a protective barrier against environmental insults. Corneal diseases are among the leading causes of blindness and visual impairment worldwide, representing a substantial global health burden. A wide range of inherited, acquired, and iatrogenic conditions, including corneal dystrophies, infections, neovascularization, stromal scarring, corneal haze, dry eye disease (DED), keratoconus, and traumatic injuries, can disrupt corneal homeostasis, impair tissue integrity, and ultimately result in vision loss [1,2].

Corneal transplantation is the current standard for visual restoration, with a significant majority of patients experiencing improved vision [3]. Despite advancements such as lamellar transplant procedures, which reduce the risk of rejection, approximately 25% of patients still encounter graft rejection within the first five years [4]. This risk escalates in cases of infectious keratitis and trauma, leading to a higher likelihood of transplantation failure. While topical steroids can mitigate early rejection episodes, severe cases may result in transplant failure, necessitating additional graft procedures and a greater supply of donor corneal tissue [5]. Consequently, there is an urgent need for alternative therapies as corneal diseases remain a major cause of vision impairment.

Gene therapy holds significant promise for addressing various corneal disorders by modulating gene expression to treat diseases or injuries. This approach encompasses gene augmentation, gene silencing, gene replacement, and precision genome editing to restore or modulate disease-associated molecular pathways. The unique accessibility and relative immune privilege of the cornea make it an ideal target for localized gene therapy. The core principle of gene therapy is the targeted delivery of therapeutic genes to corneal tissues, thereby maximizing therapeutic efficacy while minimizing systemic adverse effects [6]. Consequently, gene therapy is poised to have a substantial therapeutic impact on inherited and acquired corneal disorders, offering a potential alternative to surgery.

Recent advances in precision genome editing, vector engineering, and targeted ocular gene delivery have substantially expanded the therapeutic potential of gene therapy for corneal diseases [7]. These developments offer new opportunities to overcome the limitations of conventional pharmacological treatments and corneal transplantation by enabling targeted, long-lasting, and disease-modifying interventions. This narrative review comprehensively summarizes recent advances in gene-editing technologies, viral and nonviral gene delivery platforms, and their therapeutic applications for inherited and acquired corneal diseases. In addition, it critically discusses the current challenges, translational barriers, and emerging opportunities for advancing the clinical translation of gene-based therapies.

## 2. Literature Search and Study Selection

This narrative review was based on a structured literature search of PubMed and Google Scholar to identify relevant publications from March 2000 through February 2026. The search strategy used combinations of the following keywords and related terms: “corneal diseases,” “corneal dystrophy,” “keratoconus,” “Fuchs endothelial corneal dystrophy,” “infectious keratitis,” “corneal neovascularization,” “corneal scarring,” “gene therapy,” “gene delivery,” “gene editing,” “CRISPR/Cas9,” “Zinc Finger Nucleases,” “TALENs,” “gene augmentation,” “gene replacement,” “gene silencing,” “viral vectors,” “AAV,” “lentivirus,” “adenovirus,” “nonviral vectors,” “nanoparticles,” and “electroporation.” Studies were considered eligible for inclusion when they addressed gene-based therapeutic approaches relevant to inherited or acquired corneal diseases, including gene-editing technologies, therapeutic gene strategies, gene-delivery platforms, molecular mechanisms, preclinical studies, or clinical applications. Original research articles and relevant review articles were considered, with priority given to studies providing mechanistic evidence, demonstrating therapeutic efficacy in relevant corneal models, describing clinically relevant delivery approaches, or reporting human clinical applications.

During screening, titles and abstracts were initially evaluated for relevance, followed by assessment of the full text when necessary. Duplicate publications and studies that were unrelated to corneal diseases or gene-based therapeutic approaches, lacked sufficient information relevant to the objectives of this review, or focused exclusively on non-ocular tissues were excluded. Representative studies were selected based on their scientific relevance, novelty, therapeutic significance, and contribution to understanding gene therapy mechanisms, delivery strategies, and clinical translation in corneal diseases. Additional relevant publications were identified through manual screening of the reference lists of selected articles to incorporate important studies and emerging developments that complemented the database search.

## 3. Cornea: Structure and Function

The cornea, a transparent, avascular, convex structure located in front of the iris and pupil, allows light to enter the eye and contributes significantly to its refractive power. It comprises five layers: the epithelium, Bowman’s layer, stroma, Descemet’s membrane, and endothelium (Figure 1A,B). The cornea develops around gestational day 33, with the epithelium originating from the surface ectoderm and the stromal and endothelial layers derived from neural crest-derived mesenchymal cells [8,9].

The human corneal epithelium, approximately 50 μm thick, consists of 4–6 layers of nonkeratinized stratified squamous epithelial cells. It is composed of three cell layers: superficial (squamous) cells, wing cells, and basal cells [10]. Superficial cells, located in the outermost layers, possess features that maximize their interaction with the tear film and form tight junctions that create a protective barrier against pathogens. Wing cells provide additional support through tight junctions. Basal cells attach to the underlying basement membrane via hemidesmosomes, forming an anchoring complex that connects the epithelium to the underlying stroma [11].

Bowman’s layer, situated between the corneal epithelium and stroma, serves as a supportive structure. Its smooth anterior surface adjoins the epithelial basement membrane, whereas its posterior surface merges with the stroma. It consists of collagen fibrils within an extracellular matrix (ECM), primarily comprising collagen types I, III, V, VI, and XII [12]. Despite lacking regenerative capacity and thinning with age, recent advancements have enabled successful transplantation of Bowman’s layer from cadaveric donors to treat corneal ectasia and severe scarring [13].

The corneal stroma constitutes approximately 90% of the corneal thickness and provides stability, transparency, and tensile strength. Its structure and function heavily rely on the composition of the ECM, particularly collagen types I, III, V, XII, XIV, and various proteoglycans, including decorin and lumican. Collagen type I, the most abundant collagen in the stroma, forms bundles and lamellae arranged in a highly organized lattice, thereby providing mechanical tensile strength. Keratocytes maintain this organization. The precise alignment and orientation of collagen fibrils and lamellae are crucial for optimal stromal function [14,15,16].

Descemet’s membrane, a dense, transparent layer, separates the posterior corneal stroma from the endothelium. It is primarily produced by the corneal endothelium during gestation, and keratocytes may contribute to its repair. Structurally, it consists of collagen types IV, VII, and VIII, facilitating adhesion between the endothelium and stroma while maintaining corneal integrity, transparency, and hydration [17,18].

The endothelium, a single layer of hexagonal cells lining the posterior cornea, transports water from the stroma to maintain corneal transparency. It relies on the sodium–potassium adenosine triphosphatase (Na^+^/K^+^-ATPase) pump and bicarbonate-dependent magnesium adenosine triphosphatase (Mg^2+^-ATPase) to regulate corneal hydration [19]. Its limited regenerative capacity results in compensatory enlargement and migration of adjacent cells following injury. A decrease in endothelial cell density leads to excessive stromal hydration, resulting in corneal edema and loss of transparency [20,21].

## 4. Clinical Treatment Options Currently Available

The management of corneal diseases depends on the underlying etiology, anatomical location, and disease severity. Current treatment strategies include pharmacological therapies, minimally invasive procedures, and surgical interventions aimed at alleviating symptoms, preserving corneal integrity, and restoring visual function. Topical lubricants, corticosteroids, antibiotics, antivirals, immunomodulatory agents, and anti-angiogenic therapies are commonly used to control ocular surface inflammation, infection, and neovascularization, whereas surgical procedures are generally reserved for advanced or refractory disease [22,23].

For structural corneal disorders, phototherapeutic keratectomy (PTK) is widely used to treat superficial corneal opacities and selected corneal dystrophies. In progressive keratoconus, corneal collagen cross-linking (CXL) remains the standard treatment for halting disease progression and may be combined with adjunctive procedures such as topography-guided photorefractive keratectomy (PRK) or intracorneal ring segment implantation to improve visual outcomes. Patients with advanced corneal scarring or ectasia may require specialty contact lenses or corneal transplantation. Depending on the depth and extent of corneal involvement, visual rehabilitation can be achieved through anterior or posterior lamellar keratoplasty or penetrating keratoplasty. These procedures remain the standard surgical options for restoring corneal transparency and visual function [24,25,26].

## 5. Current Challenges in the Treatment of Corneal Diseases

While surgical interventions offer efficacy in managing such conditions, they present notable clinical and logistical hurdles. PTK stands as a valuable and efficient method, employing excimer laser ablation to address superficial corneal opacities stemming from infections, trauma, or dystrophy [27]. Nonetheless, there exists a risk of primary pathology recurrence following the procedure, particularly evident in cases of corneal dystrophies. Post-procedural complications may include induced refractive errors, infections, and corneal scarring [28].

Corneal transplantation stands as a cornerstone in treating various corneal conditions, with lamellar techniques significantly enhancing graft outcomes [29]. Despite the increasing adoption of these transplantation methods, significant challenges persist. A primary concern in eye banking and transplantation is the limited availability of viable corneal tissue for grafting [30]. The demand for corneal tissue far exceeds the available supply, with a substantial disparity of more than fourfold between required and available tissues [5]. Moreover, potential donor tissues are often rejected during screening due to concerns about transmissible diseases, including human immunodeficiency virus (HIV), hepatitis B virus (HBV), and hepatitis C virus (HCV) [31]. Additionally, over 50% of the global population lacks access to the requisite surgical expertise essential for successful corneal transplantation [1].

Another significant hurdle in corneal transplantation is the potential for graft rejection and failure. While the early survival rate of full-thickness corneal transplants exceeds 70% at one year post-surgery, this rate declines to less than 50% after five years, even in primary grafts [32]. Repeat and high-risk grafts exhibit even lower survival rates. Lamellar transplantation techniques like deep anterior lamellar keratoplasty (DALK), Descemet stripping endothelial keratoplasty (DSEK), and Descemet membrane endothelial keratoplasty (DMEK) present reduced risk of rejection and superior surgical and visual outcomes, particularly in low-risk grafts [33]. However, in high-risk grafts, the loss of corneal immune privilege due to preexisting conditions complicates treatment, often necessitating long-term immunomodulation with associated complications and variable outcomes [33]. In developing countries, challenges related to the cost of procedures, long-term follow-up, and availability of surgical expertise further compound the issue [34].

Collectively, these limitations underscore the need for innovative therapeutic strategies that target the underlying molecular mechanisms of corneal diseases rather than simply managing their clinical manifestations. In this context, gene therapy has emerged as a promising approach capable of providing targeted, long-lasting, and potentially disease-modifying treatment for both inherited and acquired corneal disorders.

## 6. Gene Therapy Technologies and Strategies for Corneal Diseases

Gene therapy has emerged as a promising therapeutic strategy for both inherited and acquired corneal diseases by enabling precise modulation of disease-associated genes and molecular pathways. Recent advances in genome editing, vector engineering, and targeted ocular delivery have substantially expanded the potential of gene-based therapies for preventing disease progression, promoting tissue regeneration, and restoring visual function. This section provides an overview of the key technologies and strategies underlying corneal gene therapy, including gene editing, gene delivery systems, and routes of vector administration.

### 6.1. Gene Editing Strategies

Recent advancements in gene editing offer promising opportunities for treating corneal diseases by precisely modifying the genome. This technology enables the development of advanced gene therapies and provides insights into the complex molecular mechanisms involved in these conditions. Gene-editing technologies such as zinc finger nucleases (ZFNs), transcription activator-like effector nucleases (TALENs), and clustered regularly interspaced short palindromic repeats (CRISPR)-based systems enable efficient and high-throughput genome engineering, revolutionizing the study and potential treatment of ocular disorders, including corneal diseases [35].

#### 6.1.1. Zinc Finger Nucleases (ZFNs)

ZFNs represent an initial generation of gene editing tools. They are synthetic chimeric proteins combining the nonspecific endonuclease cleavage domain from the *Flavobacterium okeanokoites* (FokI) restriction enzyme with the specific DNA-binding domain of eukaryotic zinc finger proteins (ZFPs) [36]. Each zinc finger motif typically recognizes a three-base-pair DNA sequence, and multiple zinc finger modules can be assembled to target specific genomic loci with high specificity. Because the FokI nuclease is catalytically active only as a dimer, two ZFN monomers are designed to bind opposite DNA strands at adjacent target sites, allowing dimerization of the FokI domains and the induction of a site-specific DNA double-strand break (Figure 2).

Following DNA cleavage, the induced double-strand break is repaired through one of two endogenous cellular repair pathways. In the absence of a donor DNA template, repair occurs predominantly through non-homologous end joining (NHEJ), which frequently introduces insertions or deletions that disrupt gene function. In contrast, the presence of a homologous donor DNA template enables homology-directed repair (HDR), allowing precise gene correction, insertion, or replacement [37].

Although ZFNs demonstrated the feasibility of targeted genome editing and laid the foundation for subsequent genome-editing technologies, their widespread application has been limited by the complexity of protein engineering, high development costs, and potential off-target effects relative to newer platforms such as TALENs and CRISPR/Cas systems [38].

#### 6.1.2. Transcription Activator-like Effector Nucleases (TALENs)

Transcription activator-like effectors (TALEs) are DNA-binding proteins originally derived from plant-pathogenic bacteria and can be engineered for sequence-specific genome editing. TALEs contain tandem repeat domains with variable amino acid residues at the 12th and 13th amino acid positions, known as repeat-variable diresidues (RVDs), which determine their DNA-binding specificity. Structural studies have demonstrated that the 13th amino acid residue directly recognizes the target nucleotide, whereas the 12th amino acid residue contributes to stabilization of the protein–DNA interaction [39].

Depending on the RVD composition, TALEs recognize different nucleotides: NI recognizes adenine (A), HD recognizes cytosine (C), NN or NK recognizes guanine (G), and NG recognizes thymine (T). By assembling multiple TALE repeats, virtually any DNA sequence can be specifically targeted. Fusion of engineered TALE DNA-binding domains with the FokI endonuclease generates TALENs, which function as dimers to introduce site-specific DNA double-strand breaks (Figure 3). Like ZFNs, these DNA breaks are repaired through NHEJ or HDR, enabling targeted gene disruption or precise genome modification. Compared with ZFNs, TALENs offer greater design flexibility, improved target specificity, and simpler protein engineering, resulting in reduced off-target effects. However, their relatively large size and limited delivery efficiency remain important limitations for their clinical translation and in vivo therapeutic applications [40].

#### 6.1.3. Clustered Regularly Interspaced Short Palindromic Repeats (CRISPR)/CRISPR-Associated Protein 9 (Cas9)

CRISPR/Cas9 is currently the most versatile and widely used genome-editing platform owing to its simplicity, efficiency, and programmability. It has been extensively applied for targeted gene disruption, gene correction, functional genomics, disease modeling, and the development of novel therapeutic strategies [41]. Beyond genome editing, CRISPR/Cas systems have also been adapted for epigenome editing, transcriptional regulation, chromosomal imaging, molecular diagnostics, and biosensing applications [42].

The CRISPR/Cas9 system consists of the Cas9 endonuclease and a single-guide RNA (sgRNA), which directs Cas9 to a complementary target DNA sequence adjacent to a protospacer adjacent motif (PAM). Recognition of the PAM sequence is essential for target DNA binding and cleavage. Upon target recognition, Cas9 introduces a site-specific double-strand break, which is subsequently repaired through endogenous cellular DNA repair mechanisms (Figure 4). In the absence of a donor DNA template, repair occurs primarily through NHEJ, often resulting in insertions or deletions that disrupt gene function. Alternatively, in the presence of a homologous donor DNA template, HDR enables precise gene correction, insertion, or replacement [43,44].

Compared with ZFNs and TALENs, CRISPR/Cas9 offers several advantages, including simpler guide RNA design, greater targeting flexibility, higher genome-editing efficiency, and lower development costs. These features have established CRISPR/Cas9 as the preferred genome-editing platform for basic research and translational applications, including emerging gene therapy approaches for inherited and acquired corneal diseases. Nevertheless, challenges such as off-target editing, PAM sequence requirements, immune responses to Cas proteins, and efficient in vivo delivery remain major obstacles to the safe and effective clinical translation of CRISPR/Cas9-based therapies [45].

### 6.2. Therapeutic Gene Strategies

The therapeutic success of gene therapy depends not only on efficient gene-editing technologies but also on the selection of an appropriate therapeutic strategy based on the underlying disease mechanism. Depending on the genetic basis and clinical characteristics of the disorder, gene therapy may aim to restore normal gene function, suppress the expression of a pathogenic gene, replace or correct disease-causing mutations, or overcome vector-related delivery limitations. Accordingly, several complementary gene therapy strategies, including gene augmentation, gene silencing, and dual-vector systems, have been developed to enable precise and targeted interventions for inherited and acquired corneal diseases.

#### 6.2.1. Gene Augmentation

Gene augmentation therapy is a direct method involving the transfer of a functional therapeutic gene into affected cells to re-establish the expression of a deficient gene (Figure 5A) [46]. This method primarily applies to recessive genetic disorders. For effective augmentation, the introduced transgene must produce sufficient levels of the normal protein to meet physiological requirements. Moreover, treatment should be initiated at a non-terminal stage, considering the status of the affected tissue and disease progression. To mitigate unintended transgene expression and its potential pathogenic impact on surrounding cells, precise regulation of transgene expression is paramount for gene augmentation strategies. Achieving appropriate transgene regulation entails the design of cell- or tissue-specific promoter sequences to control the expression of the therapeutic transgene. For instance, nuclear factor kappa-light-chain-enhancer of activated B cells (NF-κB)-responsive promoter sequences have been employed to induce transgene expression in immune organs during inflammation [47].

In gene augmentation therapy targeting secretory proteins, a prevalent strategy involves directing therapeutic gene delivery to a remote tissue or cell rather than to the specific affected cell type or tissue. This facilitates the production of the deficient therapeutic secretory protein, thereby ameliorating the disease phenotype. This strategy is particularly applicable under the following circumstances: (i) when transduction of affected cells fails to generate a physiologically significant quantity of secreted transgene protein; (ii) when the targeted gene delivery site is susceptible to additional damage during the delivery process; and (iii) when the delivery site is highly exposed to the host immune system, potentially resulting in an immune response directed against the transgene [48]. To the best of our knowledge, such an approach has not yet been reported in corneal gene therapy.

#### 6.2.2. Gene Silencing

Gene augmentation therapy is generally insufficient to treat autosomal dominant disorders because the mutant allele continues to produce a pathogenic protein. To ensure the efficacy of augmentation therapy, suppression of mutant gene expression is often required beforehand. This can be accomplished by administering small double-stranded non-coding RNA molecules specifically engineered to induce RNA interference (RNAi), thereby silencing the mutant gene (Figure 5B) [49]. RNAi facilitates post-transcriptional gene suppression through enzymatic degradation of RNA molecules that are complementary to the target sequence. This process is orchestrated by a multi-protein complex known as the RNA-induced silencing complex (RISC) [50]. Small interfering RNAs (siRNAs) have been experimentally assessed in diverse corneal diseases, encompassing conditions such as Meesmann epithelial corneal dystrophy (MECD), wound healing disturbances, and neovascularization [51,52].

Among gene silencing approaches, the most widely investigated strategy involves employing antisense oligonucleotides (AONs). These AONs, typically short single-stranded DNA or RNA molecules, bind to complementary mRNA, thereby impeding translation through modulation of pre-mRNA splicing [53]. The first AON approved by the U.S. Food and Drug Administration (FDA) was fomivirsen (marketed as Vitravene), which was used for the treatment of cytomegalovirus retinitis [54]. In corneal applications, the first Phase I clinical trial evaluated the safety and tolerability of topically administered aganirsen, an AON targeting insulin receptor substrate-1 (IRS-1) for the treatment of corneal neovascularization [55]. Aganirsen demonstrated therapeutic efficacy by reducing the need for corneal transplantation [56]. Additionally, GS-101, an AON, exhibited notable anti-angiogenic properties, leading to substantial regression of corneal neovascularization [57]. Although gene silencing strategies have demonstrated promising therapeutic outcomes, they often fail to achieve complete suppression of target gene expression. Furthermore, significant limitations, including off-target effects and the potential toxicity of RNA interference-based therapeutics, continue to limit their broader clinical translation.

#### 6.2.3. Dual Vectors

One of the major limitations of recombinant adeno-associated virus (rAAV)-based gene therapy is its limited packaging capacity of approximately 4.7 kb, which restricts the delivery of large therapeutic genes, such as programmed death ligand 1 (PD-L1), frequently employed in investigations concerning corneal graft rejection. Various approaches have been devised to overcome this limitation by exploiting the head-to-tail concatemerization of the AAV genome [58]. One such approach is the overlapping dual-vector strategy, wherein effective reconstitution and transgene expression require the utilization of two AAV vectors, each harboring one half of a large therapeutic gene. Upon co-infection of the target cell by both vectors, the two halves are reconstituted through the inherent capacity of AAV genomes for concatemerization via intermolecular recombination [59].

A trans-splicing dual-vector strategy has been developed to enhance the packaging capacity of the rAAV system. This strategy incorporates a splice donor at the 3′ end of one segment of the target gene within one vector and a splice acceptor at the 5′ end of the other segment of the transgene, facilitating the reconstitution of full-length messenger RNA (mRNA) through head-to-tail concatemerization [60]. Another dual-vector strategy, known as the hybrid approach, combines features of the overlapping and trans-splicing methods by incorporating a highly recombinogenic exogenous sequence to enhance recombination efficiency. This approach has been shown to improve transgene reconstitution and expression compared with conventional overlapping and trans-splicing dual-vector strategies in both cell culture and animal models [61,62,63].

### 6.3. Gene Therapy Delivery Methods

Gene delivery refers to the transfer of therapeutic nucleic acids, including DNA and RNA, into specific target cells for therapeutic purposes. Choosing an appropriate gene delivery system is crucial for achieving efficient, safe, and targeted gene transfer. Based on the delivery vehicle, gene delivery systems can be broadly categorized into two groups: viral vectors and nonviral vectors. Figure 6 illustrates the general mechanisms of viral and nonviral gene delivery, whereas Table 1 summarizes the representative examples, advantages, and limitations of commonly used gene delivery vectors.

#### 6.3.1. Viral Vector-Based Gene Delivery

Corneal gene therapy and research primarily rely on viral vector-based gene delivery due to the high transduction efficiency of viral vectors, their ability to efficiently deliver therapeutic genes, and their capacity to protect genetic cargo from intracellular degradation [64,65]. Viral vectors commonly utilized for gene delivery include adenovirus (AdV), herpes simplex virus (HSV), adeno-associated virus (AAV), retrovirus (RV), and lentivirus (LV). Viral gene delivery can be achieved through two approaches: in vivo, where genetic material is directly delivered into the target tissue, and ex vivo, where genetic material is introduced into cells cultured ex vivo before transplantation into the recipient [66]. The mechanisms by which these viral vectors deliver therapeutic genes and mediate transgene expression are discussed in the following sections.

##### Adeno-Associated Virus (AAV)

AAV is a small, non-enveloped virus approximately 25 nm in diameter that contains a linear single-stranded DNA (ssDNA) genome of approximately 4.7 kb. The viral genome comprises two major genes: the rep gene, which encodes proteins involved in viral genome replication, integration, and regulation of gene expression, and the cap gene, which encodes the structural capsid proteins VP1, VP2, and VP3 [67].

AAV belongs to the Parvoviridae family and the Dependoparvovirus genus and requires a helper virus, such as AdV or HSV, for productive replication. In gene therapy, recombinant AAV (rAAV) vectors are engineered by replacing the viral coding sequences with a therapeutic transgene while retaining the inverted terminal repeats required for genome packaging and expression. Consequently, rAAV vectors are replication-deficient, exhibit low immunogenicity, and efficiently transduce both dividing and non-dividing cells, making them one of the safest viral vectors for gene therapy applications [68].

The broad ocular tropism of different rAAV serotypes is attributed to their ability to bind cell surface receptors, including heparan sulfate proteoglycans (HSPGs), followed by receptor-mediated endocytosis [69]. Following cellular entry, the viral genome is released into the nucleus, where the single-stranded DNA is converted into double-stranded DNA by host cellular machinery before undergoing transcription and translation. The recombinant AAV genome predominantly persists as an episome, enabling long-term transgene expression while minimizing the risk of insertional mutagenesis. However, the relatively small packaging capacity of approximately 4.7 kb remains a major limitation of rAAV vectors [70].

Extensive studies have demonstrated that different rAAV serotypes efficiently transduce anterior segment tissues of the eye, highlighting the importance of selecting appropriate serotypes, promoters, and delivery routes to achieve cell-specific gene expression [71]. Among these, the corneal stroma has emerged as a particularly attractive target because quiescent stromal keratocytes can sustain long-term transgene expression. The first reported AAV-mediated gene therapy for corneal disease utilized the AAV2 serotype, demonstrating successful transgene delivery into rabbit corneas in vivo [72]. However, the high seroprevalence of AAV2 in humans may result in pre-existing neutralizing antibodies that reduce transduction efficiency and therapeutic efficacy.

In contrast, the AAV5 serotype exhibits significantly higher transduction efficiency than AAV2 in the cornea [71]. Topical AAV5-mediated gene delivery in rabbit models has significantly reduced corneal haze and fibrosis without detectable immunogenicity or toxicity [73]. Furthermore, AAV8 and AAV9 efficiently transduce corneal keratocytes [71,74]. To further improve gene delivery efficiency, chimeric AAV8/9 vectors combining the AAV8 capsid scaffold with the AAV9 galactose-binding domain have been developed. These vectors demonstrated widespread transgene expression in human donor corneas following intrastromal administration and exhibited superior transduction efficiency compared with either parental serotype [75].

Overall, recombinant AAV vectors remain among the most promising viral gene delivery platforms for corneal gene therapy because of their favorable safety profile, efficient ocular transduction, prolonged transgene expression, and low immunogenicity. Nevertheless, their limited packaging capacity and pre-existing immunity remain important challenges for broader clinical translation.

##### Lentivirus (LV)

LVs, classified within the Retroviridae family, are enveloped single-stranded RNA viruses measuring approximately 80–100 nm in diameter. Lentiviral transduction begins with binding of the viral envelope glycoproteins to specific host cell surface receptors, followed by fusion of the viral envelope with the host cell membrane, facilitating the entry of the viral nucleoprotein complex into the cytoplasm [76]. Subsequently, the viral RNA is reverse-transcribed into double-stranded DNA by viral reverse transcriptase. The resulting dsDNA is then transported into the nucleus through the nuclear pore complex and integrated into the host genome by viral integrase, enabling stable and long-term transgene expression [77].

One of the primary advantages of lentiviral vectors is their relatively large transgene packaging capacity, broad cellular tropism, and ability to efficiently transduce both dividing and non-dividing cells. Ex vivo gene therapy studies employing lentiviral vectors to treat corneal graft rejection and corneal fibrosis in experimental models have demonstrated efficient and sustained transgene expression in corneal epithelial cells, keratocytes, and endothelial cells, resulting in improved therapeutic outcomes [78,79].

However, lentiviral vectors also present several safety concerns, including host immune responses, random integration into the host genome, and the associated risk of insertional mutagenesis [7]. Therefore, careful vector design and comprehensive safety evaluation are essential before the clinical application of lentiviral gene therapy.

##### Adenovirus (AdV)

AdVs, classified within the Adenoviridae family, are non-enveloped, double-stranded DNA viruses. More than 50 human adenoviral serotypes have been identified, with serotypes 2 and 5 being the most commonly used for gene therapy applications [80]. AdVs possess the ability to transduce both dividing and non-dividing cells and accommodate therapeutic genes of up to approximately 30 kb, enabling efficient gene delivery to target tissues. Because AdVs remain predominantly episomal, they exhibit a low risk of insertional mutagenesis. Additionally, AdV production yields high viral titers, facilitating efficient transduction with relatively small injection volumes, making them attractive vectors for corneal gene therapy [81].

AdVs deliver therapeutic genes into host cells through receptor-mediated endocytosis by binding to the coxsackievirus and adenovirus receptor (CAR) and integrins, including αvβ3 and αvβ5, followed by clathrin-mediated internalization. Subsequently, the viral genome is released into the cytoplasm and transported into the nucleus through the nuclear pore complex, where it persists as an episome and directs transgene expression without integrating into the host genome [82].

However, a major limitation of AdVs is the transient expression of therapeutic transgenes, primarily due to epigenetic silencing of the episomal viral genome. Consequently, repeated vector administration is often required, which may increase cytotoxicity and immunogenicity [83]. Moreover, adenoviral capsid proteins are potent targets for rapid immune recognition, eliciting strong innate and adaptive immune responses [81]. Pre-existing immunity to wild-type adenoviruses in humans may further reduce the efficiency of adenoviral vector-mediated gene delivery.

##### Retrovirus (RV)

RVs, members of the Retroviridae family, were among the first viral vectors investigated for gene therapy applications. They are enveloped, single-stranded RNA viruses with a genome size of approximately 7–11 kb and contain three essential genes (gag, pol, and env) that encode structural proteins, viral enzymes, and envelope glycoproteins, respectively [81]. Following receptor-mediated membrane fusion, the viral RNA is reverse-transcribed into double-stranded DNA and integrated into the host genome by viral integrase, enabling stable and long-term transgene expression [80].

The major advantages of retroviral vectors include long-term transgene expression and relatively low immunogenicity. However, their clinical application is limited by random genomic integration, the risk of insertional mutagenesis, and their inability to efficiently transduce non-dividing cells, making them less suitable for ocular tissues such as the corneal endothelium [81].

#### 6.3.2. Nonviral Vector-Based Gene Delivery

Nonviral gene delivery methods encompass physical and chemical approaches, which are associated with lower immunogenicity, improved safety, and greater cargo flexibility than viral vectors. Physical methods include electroporation, sonoporation, photoporation, magnetofection, ballistic DNA delivery (gene gun), and direct injection of genetic material into target tissues [84]. Chemical vectors include liposomes, lipid nanoparticles (LNPs), polymers, dendrimers, and gold nanoparticles, which facilitate intracellular delivery of therapeutic nucleic acids primarily through endocytosis. Cationic liposomes and LNPs encapsulate nucleic acids to form lipoplexes, whereas polymers and dendrimers form polyplexes that enhance cellular uptake and protect genetic cargo from degradation. Gold nanoparticles have also emerged as promising nonviral carriers because of their high biocompatibility, large surface area, and efficient nucleic acid loading capacity [84,85]. In addition, polymeric nanoparticles incorporating biocompatible polysaccharides such as dextran, chitosan, and hyaluronic acid have demonstrated considerable potential for efficient ocular gene delivery [86,87].

##### Electroporation

Electroporation employs brief, high-voltage electric pulses to transiently permeabilize the cell membrane, facilitating the delivery of plasmid DNA or other therapeutic nucleic acids into target cells. This nonviral gene delivery approach has demonstrated efficient transgene delivery to both corneal endothelial cells and stromal keratocytes, with an optimal electric field strength ranging from 100 to 200 V/cm [88,89]. In vivo studies in animal models of stromal keratitis and corneal endothelial wound healing have demonstrated approximately a 1000-fold increase in corneal gene uptake compared with plasmid DNA injection alone, resulting in significant improvement of disease phenotypes [90,91,92]. However, irreversible membrane permeabilization, heat-induced tissue damage, and limited cell viability remain important limitations of electroporation [93].

##### Nanoparticles

Nanoparticles, typically ranging from 1 to 100 nm in diameter, have emerged as promising nonviral gene delivery vehicles because of their high loading capacity, favorable biocompatibility, efficient cellular uptake, and ability to overcome ocular barriers. They can deliver DNA, RNA, peptides, proteins, antibodies, and therapeutic drugs to specific ocular tissues, including the cornea, conjunctiva, sclera, and, in some cases, the retina. Nanoparticles are broadly classified into metallic, polymeric, lipid-based, and hybrid nanoparticles. Among these, polymeric nanoparticles, including those derived from albumin, chitosan, and polyethyleneimine (PEI), have demonstrated efficient transgene delivery to rodent corneas with minimal toxicity [94,95]. Hybrid nanoparticles have also shown efficient gene delivery and significant antifibrotic effects in rabbit models of corneal fibrosis without detectable adverse effects [96].

**Table 1:** Representative examples, advantages, and limitations of viral and nonviral gene delivery vectors used for corneal gene therapy.

Vector	Vector Type	Examples	Advantages	Disadvantages	Reference
	Adeno-associated virus (AAV)	AAV2, AAV5, AAV8, AAV9	Low immunogenicity, long-term gene expression, broad corneal tropism, non-pathogenic, and high safety profile	Limited cargo capacity (~4.7 kb), pre-existing neutralizing antibodies, and repeated administration may be limited	[97]
Viral	Adenovirus (AdV)	Ad5, Ad35	High transduction efficiency, large cargo capacity (up to ~36 kb), infects dividing and non-dividing cells	Strong immune response, transient gene expression, and inflammatory toxicity	[98]
	Lentivirus (LV)	HIV-derived, EIAV	Stable genomic integration, long-term expression, and relatively large cargo (~8 kb)	Risk of insertional mutagenesis, lower biosafety, and potential oncogenicity	[99]
	Retrovirus (RV)	MLV	Stable integration and sustained expression	Only infects dividing cells and insertional mutagenesis risk	[100]
	Herpes simplex virus (HSV)	HSV-1 vectors	Very large cargo capacity (>30 kb) and efficient ocular tropism	Cytotoxicity, immunogenicity, and biosafety concerns	[101]
	Lipid nanoparticles (LNPs)	Ionizable LNPs	Nonviral, low immunogenicity, large cargo capacity, suitable for mRNA and CRISPR delivery, and scalable production	Transient expression, lower delivery efficiency than viral vectors	[102]
	Polymeric nanoparticles	PLGA, PEI, Chitosan	Biocompatible, biodegradable, controlled release, and customizable	Lower transfection efficiency and possible cytotoxicity depending on polymer	[103]
Nonviral	Dendrimers	TAT, Penetratin	Simple formulation, low immunogenicity, and facilitates intracellular delivery	Limited cargo size, rapid degradation, and relatively low efficiency	[104]
	Gold nanoparticles	Functionalized AuNPs	Excellent biocompatibility, easy surface functionalization, efficient nucleic acid loading, enhanced cellular uptake, and imaging capability	Non-biodegradable, possible dose-dependent accumulation and toxicity, and limited long-term safety data	[105]

#### 6.3.3. Considerations for Vector Selection in Corneal Gene Therapy

Vector selection for corneal gene therapy should be tailored to the target cell or corneal layer, the required duration of transgene expression, therapeutic cargo size, repeat-dosing requirements, and disease stage. For corneal epithelial and limbal stem cell targets, efficient access to the ocular surface and sustained transduction of the relevant cell populations are important considerations. Lentiviral vectors may provide persistent gene expression in limbal stem cells, whereas AAV vectors can efficiently transduce corneal epithelial cells but may show limited persistence in the continuously renewing epithelium [106,107]. For stromal targets, such as keratocytes involved in corneal fibrosis or dystrophies, AAV may provide effective delivery to the stromal compartment and relatively sustained transgene expression. For corneal endothelial targets, vector selection should consider the limited proliferative capacity of endothelial cells and the need for efficient delivery to the posterior cornea; both viral and nonviral approaches have been investigated for endothelial gene delivery [91,107]. Therapeutic cargo size is another important determinant, as the relatively limited packaging capacity of AAV restricts its use for larger genes, whereas adenoviral and nonviral platforms can accommodate larger genetic payloads [7,108]. In addition, nonviral systems may be advantageous when repeated administration is anticipated because of their generally lower immunogenicity and greater manufacturing flexibility, although their comparatively lower transfection efficiency and tissue penetration remain important limitations [7,109]. Disease stage should also be considered, as gene therapy is more likely to be effective when sufficient viable target cells remain, whereas advanced disease with extensive fibrosis, limbal stem-cell depletion, or severe endothelial cell loss may require complementary regenerative or surgical approaches. Thus, vector selection should be matched to the anatomical target, biological characteristics of the disease, therapeutic cargo, and intended duration of treatment [7,81].

### 6.4. Routes of Administration for Corneal Gene Delivery

The route of administration is a critical determinant of the efficacy, safety, and tissue specificity of corneal gene therapy. Owing to the unique anatomical features of the cornea, including its accessibility, transparency, and relative immune privilege, localized ocular delivery is generally preferred over systemic administration because it enables targeted gene transfer while minimizing systemic exposure and off-target effects. Depending on the target corneal layer and therapeutic objective, gene delivery can be achieved through topical administration, subconjunctival injection, intrastromal injection, intracameral injection, intravitreal injection, and, in selected cases, systemic administration (Figure 7) [6,35,91,108,110].

Among these approaches, topical administration is the least invasive and provides excellent patient compliance; however, its gene delivery efficiency is limited by tear turnover, blinking, nasolacrimal drainage, and the corneal epithelial barrier, which reduce vector retention and penetration. Subconjunctival injection provides prolonged local bioavailability and facilitates diffusion of therapeutic agents into the cornea without directly penetrating the tissue. Intrastromal injection enables efficient and localized gene transfer to stromal keratocytes, making it particularly suitable for treating stromal disorders such as corneal fibrosis, keratoconus, and inherited corneal dystrophies. Intracameral injection primarily targets the corneal endothelium and anterior chamber and has been investigated for the treatment of endothelial dysfunction and prevention of corneal graft rejection. In contrast, intravitreal and systemic administration have relatively limited applications in corneal gene therapy because they do not directly target corneal tissues and may increase the risk of systemic exposure and off-target effects [6,91,111].

## 7. Therapeutic Applications of Gene Therapy in Corneal Diseases

Corneal diseases encompass a diverse range of inherited and acquired disorders that ultimately compromise corneal transparency and visual function. Recent advances in gene therapy have enabled the development of targeted therapeutic strategies to correct disease-causing genetic defects, modulate pathogenic signaling pathways, and promote corneal repair and regeneration. An overview of gene therapy applications in major corneal diseases is illustrated in Figure 8, and representative preclinical and clinical studies are summarized in Table 2.

### 7.1. Corneal Fibrosis

Corneal injury can induce the formation of opaque scars due to the activation of myofibroblasts and the abnormal accumulation of excess ECM components, primarily mediated by transforming growth factor-β1 (TGF-β1), ultimately leading to corneal fibrosis [112].

One of the earliest studies investigating gene therapy for corneal fibrosis employed a cyclin G1 gene variant (an anti-proliferative, dominant-negative mutant cyclin G1 construct) delivered via an RV vector by topical application in rabbit models following PTK [113]. This treatment inhibited keratocyte proliferation and reduced ECM deposition without affecting epithelial cell proliferation.

Subsequently, Saika and colleagues utilized bone morphogenetic protein 7 (BMP-7) delivered via an AAV vector and evaluated its therapeutic efficacy in a murine alkali burn model. Topical AAV-BMP7 treatment significantly reduced corneal scarring after 20 days. Using the same delivery platform, the authors also demonstrated that AAV-mediated peroxisome proliferator-activated receptor-γ (PPAR-γ) gene therapy suppressed profibrotic signaling and promoted corneal epithelial healing following alkali burn injury [114,115].

Wang et al. employed the Smad7 gene delivered via an LV vector in a rat PRK model [116]. Topical LV-Smad7 therapy inhibited TGF-β/Smad signaling, thereby significantly reducing postoperative corneal fibrosis. Similarly, topical administration of AAV5-Smad7 significantly attenuated post-PRK corneal scarring in rabbit corneas [73].

Additional studies have further demonstrated the therapeutic potential of AAV-mediated gene delivery for corneal fibrosis. Intrastromal delivery of the human leukocyte antigen-G (HLA-G) gene using an AAV8G9 vector in a rabbit model of injury-induced corneal fibrosis reduced α-smooth muscle actin (α-SMA) expression and attenuated stromal fibrosis [117]. Likewise, AAV-mediated decorin gene therapy suppressed TGF-β-driven fibrotic responses and significantly reduced corneal fibrosis in preclinical models [118,119]. More recently, AAV5-mediated delivery of the inhibitor of differentiation 3 (Id3) gene markedly reduced alkali burn-induced corneal fibrosis in rabbit eyes by suppressing myofibroblast differentiation, decreasing α-SMA, fibronectin, and collagen I/III expression, and restoring corneal transparency without impairing normal corneal wound healing or causing ocular toxicity [120]. Furthermore, studies evaluating AAV5-mediated gene delivery in canine and equine corneas demonstrated efficient transgene expression and favorable safety profiles, supporting the translational potential of AAV5 as a gene delivery platform for future corneal gene therapy applications [121,122].

### 7.2. Corneal Epithelial Wound Healing

When the corneal epithelium, stroma, or endothelium sustains injury, a wound-healing response is initiated, with the nature of the response depending on the depth of the injury. Additionally, microbial infections can trigger immune responses that further influence the wound healing process. In cases of epithelial injury that does not extend into the stroma, such as superficial epithelial abrasions or mild infections, keratocyte death is minimal, and normal tissue architecture is restored through epithelial regeneration while myofibroblasts undergo apoptosis. In contrast, moderate to severe corneal injuries or infections induce excessive ECM deposition, resulting in corneal haze, scarring, and loss of transparency [123,124,125,126].

Several gene therapy approaches have been investigated to accelerate corneal epithelial wound healing and restore corneal transparency. Zagon et al. delivered the opioid growth factor receptor (OGFr) gene using a gold particle gene gun in rats, demonstrating significantly accelerated epithelial wound healing in vivo [127]. Similarly, delivery of the B-cell lymphoma-extra-large (Bcl-xL) gene via polymeric micelles reduced apoptosis and promoted epithelial wound healing following corneal epithelial debridement in a mouse model [128].

Saghizadeh et al. conducted an ex vivo study using the tyrosine-protein kinase Met (c-Met) gene delivered via an AAV vector in human diabetic corneal organ culture [129]. AAV-mediated c-Met gene delivery enhanced c-Met expression, normalized selected diabetic markers, and reduced epithelial wound healing time by approximately twofold compared with control corneas.

Subsequent studies demonstrated that AAV-mediated delivery of cathepsin F and matrix metallopeptidase 10 (MMP-10) short hairpin RNA (shRNA) significantly enhanced corneal epithelial wound healing while restoring the expression of diabetic markers and putative limbal stem cell markers in human diabetic corneal organ culture [130]. These findings were further confirmed in a subsequent ex vivo study, which demonstrated that combined delivery of c-Met, cathepsin F, and MMP-10 promoted epithelial regeneration and improved corneal wound healing [131].

More recently, Kramerov et al. introduced microRNA-409 and cathepsin F genes into human corneal organ culture using nano bioconjugates with antisense ASOs [132]. Their study indicated that these genes not only restored expression of diabetic markers and putative stem cells but also normalized epithelial wound healing in the cornea.

### 7.3. Corneal Allograft Rejection

Corneal grafting involves the replacement of either the entire cornea (penetrating keratoplasty) or selected corneal layers (lamellar keratoplasty) to restore vision in patients with corneal injury or disease. Despite significant advancements in corneal transplantation over the past two decades, immune-mediated graft rejection remains one of the leading causes of graft failure. Consequently, considerable efforts have focused on developing strategies to prolong graft survival, with gene therapy emerging as a promising approach for modulating alloimmune responses and promoting immune tolerance [133].

AAV-mediated interleukin-10 (IL-10) gene delivery in an ex vivo sheep corneal organ culture prolonged corneal allograft survival by approximately 2.7-fold compared with controls [134]. Similarly, delivery of the IL-12 p40 gene using an AAV vector promoted local intraocular expression of IL-12 p40 and extended graft survival [135]. In contrast, IL-4 gene delivery induced inflammation, eosinophilia, and accelerated graft rejection. Furthermore, IL-12 p40 gene therapy failed to improve graft survival in a rat model, suggesting species-specific differences and potential limitations associated with endogenous IL-12p35 interactions [136].

Additional studies have investigated anti-inflammatory and antiapoptotic gene therapies to further improve graft survival. Ex vivo transduction of the viral IL-10 gene using liposomes and an AAV vector did not significantly prolong graft survival, whereas systemic AAV-mediated IL-10 gene delivery significantly extended graft survival in rats [137]. Moreover, IL-10 gene transfer using LV and AdV vectors increased graft survival in human and sheep corneal organ cultures, with AAV-mediated IL-10 delivery demonstrating superior efficacy [134,138]. Subsequent studies further demonstrated that both AAV and LV vectors efficiently delivered IL-10 to limbal graft tissues ex vivo, resulting in delayed corneal allograft rejection [139]. Likewise, LV-mediated PD-L1 gene therapy suppressed inflammatory responses by reducing lymphocyte infiltration and decreasing IL-6 and interferon-γ (IFN-γ) expression, thereby significantly improving graft survival [140].

Gene therapy approaches targeting apoptosis have also demonstrated promising therapeutic effects. LV-mediated delivery of p35 and Bcl-xL genes in an ex vivo human corneal organ culture prevented corneal endothelial cell apoptosis and significantly prolonged graft survival [141,142]. Comparative studies evaluating Bcl-2, Bcl-xL, survivin, and p35 identified Bcl-xL as the most effective antiapoptotic gene for protecting corneal endothelial cells [143]. Furthermore, LV-mediated p35 gene transfer reduced CD4^+^ T-cell infiltration and attenuated immune-mediated graft rejection following corneal transplantation [144].

### 7.4. Corneal Neovascularization

The primary determinant of corneal transparency lies in its avascularity and angiogenic privilege. However, various pathological insults, including corneal graft rejection, chemical injuries, pterygium, congenital disorders, thermal injuries, infections (fungal, parasitic, bacterial, or viral), trauma, and hypoxia, can provoke severe injuries or stimulate pro-angiogenic factors, ultimately leading to corneal neovascularization [145,146,147,148,149,150,151,152]. The presence of blood vessels compromises the cornea’s immune privilege, exacerbates inflammation, and significantly increases neovascularization through positive feedback mechanisms [153]. Consequently, considerable efforts have focused on developing gene therapy approaches to inhibit pathological angiogenesis while preserving corneal transparency.

Several studies have targeted vascular endothelial growth factor (VEGF) signaling using gene therapy [154,155,156]. Lai et al. utilized the Fms-related receptor tyrosine kinase 1 (Flt-1) gene delivered by an AAV2 vector in a rat model, demonstrating that anterior chamber administration significantly reduced corneal neovascularization following corneal cauterization [157]. Similarly, AAV-mediated Flt-1 gene therapy effectively inhibited corneal neovascularization in rats [158]. Furthermore, nonviral delivery of the Flt-1 gene using polyplex micelles also suppressed corneal neovascularization in mice [159].

Multigene and antiangiogenic gene therapies have also demonstrated promising therapeutic efficacy. Chen et al. delivered Flk-1, endostatin, and Tie2 genes using an RV vector via subconjunctival injection, resulting in significant inhibition of corneal neovascularization in mice [160]. Likewise, AAV-mediated delivery of endostatin and angiostatin genes significantly suppressed corneal neovascularization in silver nitrate-cauterized and alkali burn-induced animal models, respectively [161,162].

Parker et al. evaluated LV-mediated delivery of endostatin and angiostatin genes in rabbit corneas ex vivo, demonstrating sustained transgene expression, reduced inflammatory cell infiltration, and inhibition of corneal neovascularization [163]. Similarly, VEGF-targeted gene therapy using antisense VEGF RNA delivered by AAV vectors or VEGF-A-targeting shRNA delivered by nanoparticles effectively downregulated VEGF expression and inhibited pathological corneal neovascularization [164,165].

Additional antiangiogenic strategies have further expanded the therapeutic potential of gene therapy. Kuo et al. demonstrated that subconjunctival delivery of a plasmid encoding pigment epithelium-derived factor (PEDF) using a synthetic amphiphile interaction-18 vector significantly inhibited corneal neovascularization in rats [166]. Mohan et al. demonstrated that AAV5-mediated decorin gene therapy significantly reduced corneal neovascularization following corneal epithelial debridement in rabbits by downregulating monocyte chemoattractant protein-1 (MCP-1), VEGF, and angiopoietin while upregulating PEDF expression [155]. Likewise, lipoplex-mediated delivery of human GABPα and GABPβ genes transiently suppressed corneal neovascularization, although the therapeutic effect gradually declined after two weeks [167]. Furthermore, AAV-mediated vasohibin-1 gene therapy effectively inhibited corneal neovascularization in alkali burn-induced mouse corneas by suppressing VEGFR2 and endogenous vasohibin-1 expression [168].

The successful translation of antiangiogenic gene therapy has also been demonstrated in clinical studies. Phase II and Phase III clinical trials evaluating GS-101 (aganirsen), an AON targeting insulin receptor substrate-1 (IRS-1), demonstrated significant inhibition of corneal neovascularization in patients with keratitis [56,57]. Moreover, intrastromal AAV8G9-mediated delivery of the HLA-G gene reduced corneal neovascularization and inflammatory cell infiltration in rabbit models of corneal injury [108,117].

More recently, novel gene regulatory approaches have focused on microRNAs and gene-silencing technologies. AAVrh.8-mediated delivery of the antiangiogenic microRNA-204 (miR-204) effectively suppressed corneal neovascularization by regulating multiple angiogenesis-associated signaling pathways in alkali burn-induced mouse corneas [169]. Similarly, LNP-mediated delivery of MMP-9 shRNA inhibited endothelial cell migration and tube formation, whereas cholesterol-modified siRNA targeting stromal cell-derived factor-1 (SDF-1) suppressed Akt signaling and significantly reduced corneal neovascularization following alkali burn injury [170,171].

Compared with currently available anti-VEGF therapies, antiangiogenic gene therapy may provide several potential therapeutic advantages for corneal neovascularization. Conventional anti-VEGF agents primarily neutralize VEGF activity and can effectively suppress pathological angiogenesis; however, their therapeutic effects may be temporary, and repeated administration may be required to maintain efficacy [172,173]. In contrast, gene-based approaches can achieve sustained local modulation of angiogenic signaling through VEGF inhibition or targeting multiple angiogenesis-associated pathways, potentially reducing the frequency of treatment and providing broader control of the angiogenic microenvironment [174,175]. For example, gene therapies targeting Flt-1, endostatin, angiostatin, PEDF, decorin, miR-204, MMP-9, or SDF-1 have demonstrated inhibition of corneal neovascularization through mechanisms that extend beyond direct VEGF neutralization [110,169,176]. However, these potential advantages should be interpreted cautiously because most corneal antiangiogenic gene-therapy studies remain preclinical, and direct head-to-head comparisons with established anti-VEGF therapies are limited [174,175]. Therefore, gene therapy should currently be considered a promising complementary or future therapeutic strategy rather than a replacement for conventional anti-VEGF treatment. Further comparative studies will be necessary to determine whether gene-based approaches can provide more durable, effective, and clinically meaningful control of corneal neovascularization.

### 7.5. Infectious Keratitis

Infection with HSV-1 is the leading cause of infectious viral keratitis and is characterized by recurrent corneal inflammation, stromal scarring, opacity, and progressive vision loss. Following primary infection of the cornea and orofacial tissues, HSV-1 establishes lifelong latency within the trigeminal ganglia by persisting as episomal DNA in neuronal nuclei. Periodic viral reactivation results in recurrent corneal infection and immune-mediated inflammation, making herpetic keratitis one of the most challenging corneal diseases to manage [177]. Because of the high risk of recurrence and graft failure, corneal transplantation is often not an ideal long-term treatment option for severe herpetic keratitis [178]. Consequently, gene therapy has emerged as a promising therapeutic strategy capable of suppressing viral replication, modulating host immune responses, reducing corneal scarring, and preventing recurrent disease [179].

Plasmid-mediated IL-18 gene therapy inhibited VEGF-mediated angiogenesis and reduced corneal neovascularization in experimental herpetic keratitis [180]. Several studies subsequently investigated DNA vaccine-based approaches targeting HSV-1 glycoproteins [181,182]. Inoue et al. demonstrated that topical administration of glycoprotein D-IL-2 (gD-IL-2) plasmid DNA completely inhibited stromal keratitis, although it did not significantly reduce epithelial HSV-1 lesions [182]. Subsequent investigations demonstrated that subconjunctival or hypodermal administration of the gD-IL-2 DNA vaccine enhanced cell-mediated immunity and effectively protected against stromal herpetic keratitis [183,184].

IFN-based gene therapies have also demonstrated potent antiviral activity. A series of in vivo studies by Noisakran et al. showed that plasmid-mediated IFN-α1 gene delivery significantly reduced HSV-1 viral load in both the cornea and trigeminal ganglia while decreasing viral antigen expression and inflammatory cell infiltration [185,186]. Likewise, plasmid-mediated IFN-β gene therapy reduced HSV-1 replication in both in vitro and in vivo experimental models [187,188].

Additional vaccine-based gene therapies have further demonstrated encouraging therapeutic efficacy. A gB1 DNA vaccine administered systemically protected rabbits against HSV-1 infection by preventing encephalitis and reducing ocular disease through the induction of neutralizing antibody responses, whereas local administration failed to produce comparable protection [189]. Similarly, intramuscular administration of a multivalent DNA vaccine encoding HSV-1 glycoproteins gB, gC, gD, gE, and gI (5gP DNA) significantly reduced ocular viral replication, alleviated blepharitis, and decreased viral latency in a mouse model [190].

More recently, Amrani et al. developed an AAV-mediated dual-target CRISPR/Cas9 system targeting the essential HSV-1 genes ICP0 and ICP27. In a latent rabbit model of herpetic keratitis, CRISPR/Cas9-mediated gene editing significantly reduced viral shedding, while intravenous administration of an AAV9-CRISPR/Cas9 vector eliminated viral shedding in 92% of treated eyes and markedly reduced HSV-1 expression in the trigeminal ganglia, highlighting the potential of CRISPR/Cas9 to eradicate both active corneal infection and latent viral reservoirs [191].

Building upon these findings, an inducible dual AAV-based CRISPR/Cas9 platform combined with neutralizing antibody therapy further enhanced antiviral efficacy against HSV-1. In a mouse model of herpetic stromal keratitis, retro-orbital administration of AAVrh10-ALICE-SaCas9 together with AAV1-ALICEAb induced CRISPR-mediated cleavage of the essential viral gene ICP4 while simultaneously promoting the secretion of HSV-1-neutralizing antibodies. This combinatorial strategy significantly reduced viral titers in the cornea, trigeminal ganglia, and brain, highlighting the therapeutic potential of integrating CRISPR-mediated genome editing with immunomodulation for the treatment of refractory herpetic keratitis [192].

### 7.6. Genetic Corneal Dystrophies

Corneal dystrophies represent a rare and diverse group of inherited disorders characterized by the progressive accumulation of abnormal material within one or more corneal layers, ultimately leading to visual impairment or blindness [193]. Current treatment options are largely limited to symptomatic management and corneal transplantation, which are associated with recurrent disease, limited donor tissue availability, and the risk of graft rejection. Consequently, gene therapy has emerged as a promising strategy for correcting the underlying genetic defects through gene editing, gene silencing, and gene augmentation approaches.

MECD is caused by mutations in the keratin 12 (KRT12) gene, which encodes a cornea-specific intermediate filament protein. An in vitro study employing KRT12-Leu132Pro-specific siRNA in human limbal epithelial cells derived from MECD patients demonstrated selective suppression of the mutant KRT12 allele, highlighting the therapeutic potential of mutation-specific gene silencing [194]. Subsequently, CRISPR/Cas9-mediated genome editing targeting a single-nucleotide polymorphism-derived PAM successfully reduced mutant KRT12 mRNA expression in vitro. In vivo intrastromal administration in a humanized mouse model further induced frameshift mutations in the mutant allele through NHEJ, providing proof-of-concept for CRISPR/Cas9-based therapy for MECD [195].

TGF-β-induced (TGFBI)-associated corneal dystrophies, including lattice corneal dystrophy (LCD) and granular corneal dystrophy (GCD), are caused by mutations in the TGFBI gene. The Arg124Cys (R124C) mutation is associated with LCD type I, whereas Arg124His (R124H) and Arg555Trp (R555W) mutations are primarily associated with GCD [196,197,198]. Mutation-specific siRNA targeting the TGFBI-R124C mutant allele effectively suppressed mutant gene expression in ex vivo limbal tissue obtained from patients with LCD type I [199]. Similarly, CRISPR/Cas9-mediated HDR successfully corrected the TGFBI-R124H mutation in human corneal keratocytes derived from patients with GCD2 without detectable off-target editing, demonstrating the feasibility of precise genome editing for TGFBI-associated corneal dystrophies [200].

Macular corneal dystrophy (MCD) is an autosomal recessive stromal dystrophy caused by mutations in the carbohydrate sulfotransferase 6 (CHST6) gene, leading to impaired keratan sulfate biosynthesis and progressive corneal stromal opacity. Recently, Basol et al. established a genetically engineered zebrafish model of MCD by disrupting the CHST6 gene. CHST6 deficiency resulted in a marked reduction in keratan sulfate proteoglycans (KSPGs) within the corneal stroma, and adult zebrafish recapitulated the major clinical features of human MCD. This novel in vivo model provides a valuable platform for investigating the molecular mechanisms underlying MCD and for evaluating future gene therapy and genome-editing strategies targeting CHST6-associated corneal dystrophy [201].

Fuchs endothelial corneal dystrophy (FECD) is a progressive endothelial disorder characterized by guttae formation in Descemet’s membrane and progressive endothelial cell dysfunction. Current treatment remains largely dependent on corneal transplantation, highlighting the need for disease-modifying therapies. Expansions of CTG trinucleotide repeats within the transcription factor 4 (TCF4) gene contribute to FECD pathogenesis by sequestering muscleblind-like splicing regulator proteins (MBNL1 and MBNL2), thereby disrupting normal mRNA splicing [202]. In addition to TCF4 repeat expansions, FECD has been associated with mutations in several genes, including collagen type VIII alpha 2 chain (COL8A2), solute carrier family 4 member 11 (SLC4A11), lipoxygenase homology domains 1 (LOXHD1), and zinc finger E-box-binding homeobox 1 (ZEB1), as well as the accumulation of DNA damage [203,204]. Notably, missense mutations in COL8A2 are responsible for early-onset FECD [205]. Uehara et al. developed an adenoviral vector co-expressing Cas9 and a guide RNA targeting the mutant COL8A2 allele and demonstrated efficient gene disruption following a single intracameral injection in a mouse model of early-onset FECD. This CRISPR/Cas9-mediated gene editing reduced mutant COL8A2 expression in corneal endothelial cells, restored endothelial pump function, and maintained long-term corneal transparency without detectable adverse effects [206].

Several gene therapy strategies have been investigated for FECD. Zarouchlioti et al. demonstrated that AON therapy targeting TCF4 repeat expansions significantly reduced nuclear RNA foci and restored normal splicing in human FECD corneal endothelial cells [207]. Likewise, CRISPR/deactivated Cas9 (CRISPR-dCas9) targeting TCF4 repeats reduced toxic nuclear RNA foci by approximately tenfold in cultured FECD endothelial cells following lentiviral or lipofection-mediated delivery [208].

Additional therapeutic approaches have focused on correcting other molecular abnormalities associated with FECD. AAV-mediated gene therapy has been proposed for solute carrier family 4 member 11 (SLC4A11)-associated FECD by restoring normal protein trafficking and plasma membrane localization [209]. Furthermore, AAV-delivered CRISPR/Cas genome editing has emerged as a promising strategy for correcting pathogenic TCF4 repeat expansions and slowing disease progression [210].

More recently, metastasis-associated lung adenocarcinoma transcript 1 (MALAT1) has been identified as another therapeutic target in FECD. AON-mediated inhibition of MALAT1 reduced its expression in ex vivo human corneal endothelial cells and in vivo following intracameral or intravitreal administration in mice, providing additional proof-of-concept for RNA-targeted therapies in FECD [211,212].

Collectively, these studies demonstrate that gene silencing, AON therapy, and CRISPR/Cas-based genome editing represent promising therapeutic strategies for the treatment of inherited corneal dystrophies by directly correcting or suppressing disease-causing genetic abnormalities.

### 7.7. Multifactorial and Polygenic Diseases

Various medical conditions are associated with reduced central corneal thickness (CCT), including keratoconus, keratoglobus, brittle cornea syndrome, Ehlers–Danlos syndrome, osteogenesis imperfecta, and myopia [213]. These disorders are influenced by complex interactions between multiple genetic variants and environmental factors, making the development of targeted gene therapies more challenging than for monogenic corneal diseases. Among them, keratoconus is the most prevalent multifactorial corneal ectatic disorder, characterized by progressive corneal thinning and protrusion. Its pathogenesis is multifactorial and involves genetic susceptibility, oxidative stress, inflammation, ECM remodeling, and environmental factors such as chronic eye rubbing [214,215,216].

Altered expression of numerous genes and dysregulation of multiple molecular pathways have been implicated in keratoconus pathogenesis, including lysyl oxidase (LOX), MMP-2, MMP-9, and multiple collagen types (I, III, IV, V, VI, and VII). In addition, family-based linkage analyses and genome-wide association studies have revealed substantial genetic heterogeneity among patients with keratoconus. Several susceptibility genes, including DOCK9, FLG, TGFBI, SOD1, ZEB1, and VSX1, have been associated with disease susceptibility and progression in different populations [217,218,219,220,221].

Although no clinically approved gene therapy is currently available for keratoconus or other multifactorial corneal diseases, advances in genome editing, RNA-based therapeutics, and targeted modulation of ECM remodeling and oxidative stress pathways offer promising future therapeutic opportunities. A better understanding of the complex genetic architecture and molecular mechanisms underlying these disorders will be essential for the development of effective gene-based therapies.

### 7.8. DED

DED is a chronic multifactorial disorder characterized by dysfunction of the lacrimal functional unit and is frequently associated with Sjögren’s syndrome and other autoimmune disorders [222]. It is characterized by tear film instability, ocular surface inflammation, and neurosensory abnormalities, which, if left untreated, may lead to corneal epithelial thinning, scarring, neovascularization, and visual impairment [223]. Approximately 6.8% of adults in the United States have been diagnosed with DED, with a higher prevalence among women (8.8%) [224]. Based on its underlying etiology, DED is broadly classified into aqueous-deficient dry eye, evaporative dry eye, or a combination of both. A severe form of aqueous-deficient DED is associated with chronic graft-versus-host disease (GVHD), in which persistent inflammation impairs lacrimal gland function and is often accompanied by blepharitis, conjunctivitis, and keratitis [225].

DED is primarily managed with artificial tears, anti-inflammatory agents, immunomodulators, and supportive therapies aimed at relieving symptoms and improving tear film stability [226]. However, these treatments do not address the underlying molecular mechanisms of the disease and often require long-term administration. Consequently, gene therapy has emerged as a promising strategy for modulating inflammation, restoring lacrimal gland function, and enhancing tear secretion.

Several preclinical studies have demonstrated the therapeutic potential of gene therapy for DED [35,227,228]. Zhu et al. reported that AdV-mediated delivery of the viral IL-10 transgene to the lacrimal gland significantly suppressed Sjögren’s syndrome-like manifestations, including reduced tear production, accelerated tear film breakup, ocular surface damage, and inflammatory responses in rabbits [229]. Similarly, AdV-mediated expression of a tumor necrosis factor (TNF) inhibitor (AdTNFRIp55-Ig) in the lacrimal glands of rabbits with autoimmune dacryoadenitis reduced inflammation and improved clinical outcomes [230]. Additional studies demonstrated beneficial therapeutic effects following AdV-mediated erythropoietin (EPO) gene delivery and AAV2-mediated aquaporin-1 (AQP1) gene transfer, both of which improved lacrimal gland function and tear secretion in experimental models [231,232].

Given the established role of immune dysregulation in DED pathogenesis, gene therapies targeting immunomodulatory pathways may provide additional therapeutic opportunities. For example, AAV-mediated delivery of immunoregulatory genes such as HLA-G and TGF-β has been proposed as a potential therapeutic strategy to suppress ocular surface inflammation and promote immune tolerance, although further preclinical and clinical investigations are required [117,162].

### 7.9. Mechanical Injuries and Chemical Burns

The cornea, as the outermost transparent tissue of the eye, is highly susceptible to mechanical trauma and chemical injuries, which can result in epithelial defects, stromal damage, inflammation, and vision impairment. Initial management focuses on immediate irrigation (for chemical burns), infection prevention, inflammation control, pain relief, and promotion of epithelial healing. However, severe injuries often lead to persistent inflammation, corneal neovascularization, fibrosis, limbal stem cell deficiency, and permanent vision loss despite conventional treatment [123,233,234,235].

In advanced cases, additional therapeutic interventions, including limbal stem cell transplantation, amniotic membrane transplantation, and keratoprosthesis, may be required to restore ocular surface integrity. Nevertheless, these approaches do not directly target the molecular pathways responsible for inflammation and fibrosis. Corneal injury induces the release of pro-inflammatory cytokines and profibrotic mediators, particularly TGF-β, which promote myofibroblast activation, ECM deposition, corneal scarring, and neovascularization. Consequently, gene therapy has emerged as a promising strategy to modulate these pathological pathways and improve corneal wound healing [119,155].

Several preclinical studies have demonstrated the therapeutic potential of gene therapy for mechanical and chemical corneal injuries [73,114,117,236]. Topical AAV5-mediated Smad7 gene therapy suppressed TGF-β/Smad signaling, leading to reduced corneal haze, fibrosis, and inflammatory cell infiltration [73]. Similarly, AdV-mediated PPAR-γ gene therapy promoted epithelial regeneration and suppressed profibrotic responses in a mouse model of alkali burn injury [6]. Furthermore, intrastromal delivery of the HLA-G gene using an AAV8G9 vector reduced corneal neovascularization and inflammatory cell infiltration, highlighting its potential as an immunomodulatory strategy for the treatment of severe corneal injuries [117].

**Table 2:** Summary of gene therapy studies for corneal diseases, including therapeutic genes, delivery systems, administration routes, experimental models, and therapeutic outcomes.

Gene Delivery System	Therapeutic Gene	Corneal Diseases	Mode of Administration	Animals	Model	Outcomes	References
AAV5	Id3	Corneal fibrosis/scarring	Intrastromal	Rabbit	In vivo	Significantly reduced corneal haze, α-SMA expression, fibronectin, collagen I/III deposition, and myofibroblast differentiation while restoring corneal transparency without impairing corneal wound healing or causing ocular toxicity.	[120]
rAAV	IDUA	MPS I-associated corneal blindness	Intrastromal	Human	Ex vivo	Effective widespread transduction led to a >10-fold supraphysiological increase in IDUA activity. No significant apoptosis was detected.	[108]
rAAV	Angiostatin	Corneal neovascularization	Subconjunctival	Rat	In vivo	Significant reduction in alkali burn-induced corneal angiogenesis.	[162]
rAAV	IDUA	MPS1-associated Corneal blindness	Intrastromal	Canine	In vivo	Rapid resolution of clouding within 1 week, sustained clarity for 25 weeks. Prevented advanced corneal changes and restored corneal transparency.	[237]
rAAV	Smad7	Corneal scarring	Topical	Rabbit	In vivo	Reduced stromal haze and fibrosis. No immunogenic or toxic reactions were detected.	[73]
rAAV	HLA-G1	Corneal inflammation	Intrastromal	Rat	In vivo	Reduction in corneal inflammation, vascularization, and fibrosis after corneal injury.	[238]
rAAV	Flt-1	Corneal neovascularization	Anterior chamber	Rat	In vivo	Following cauterization, corneal neovascularization reduced by 36% compared to control in stroma of rats.	[157]
rLV	p35	Corneal fibrosis	Topical	Mouse	In vivo	Decreased graft-induced immune response by reducing CD4+ T-cell expression in the cornea.	[144]
rLV	Endostatin/Kringle 5	Corneal neovascularization	Subconjunctival	Rabbit	In vivo	Corneas treated with rLV.E-K-5 vector showed decreased neovascularization and lower graft failure rates compared to control. Control group exhibited early and extensive neovascularization, with 5 out of 6 animals experiencing graft failure.	[239]
rLV	PD-L1	Corneal graft rejection	Topical	Rat	In vivo	Enhanced expression of PD-L1 in corneal cells contributed to prolonged survival of transplanted tissue by reducing the production of inflammatory signaling molecules.	[140]
rLV	Bcl-xL	Corneal graft rejection	Topical	Mouse	In vivo	Corneal endothelial delivery of rLV-Bcl-xL enhanced allograft survival by inhibiting endothelial cell apoptosis, resulting in significant improvement compared to controls.	[143]
LV	Endostatin: K5 fusion gene	Corneal allograft rejection	Ex vivo corneal transduction	Rabbit	In vivo	Inhibited corneal neovascularization, reduced inflammation, and prevented corneal graft failure following transplantation.	[239]
rLV	Smad7	Corneal fibrosis	Topical	Rat	In vivo	Reduced TGFβ/Smad signaling via Smad2 phosphorylation downregulation, inhibited cell proliferation, and decreased fibrotic marker expression.	[116]
LV	Angiostatin, endostatin	Corneal transplantation (high-risk graft rejection)	Ex vivo donor corneal gene modification	Rabbit	In vivo	Reduced corneal neovascularization, opacity, corneal thickness, and graft rejection. Demonstrated good safety and tolerability.	[240]
AdV	Anti-CD4 scFv	Corneal transplantation (allograft rejection)	Ex vivo corneal transduction	Rat	In vivo	Local anti-CD4 scFv expression inhibited allostimulation in vitro but did not significantly prolong corneal allograft survival following transplantation.	[241]
AdV	BMP-7	Corneal scarring	Topical	Mouse	In vivo	Reduced scarring in alkali-burned corneas after 20 days of infection.	[114]
AdV	CTLA4-Ig	Corneal allograft rejection	Intraperitoneal	Rat	In vivo	Ex vivo gene transfer modestly prolonged graft survival, whereas systemic AdCTLA4-Ig significantly prolonged graft survival and reduced T-cell infiltration and inflammatory cytokine expression.	[242]
AdV	Smad7	Corneal scarring	Topical	Mouse	In vivo	Decreased activation or inhibition of Smad3 and nuclear factor-κB signaling via RelA/p65 in burned corneal tissue.	[243]
AdV	c-Met	Corneal wound healing	Topical	Human	Ex vivo	Restored HGF signaling, normalized markers of diabetes, and hastened wound healing.	[129]
AdV	Vasohibin-1	Corneal neovascularization	Subconjunctival	Mouse	In vivo	Markedly diminished scarring in alkali-burned corneas.	[168]
AdV	PPAR-γ	Corneal fibrosis	-	Mouse	In vivo	Resulted in reduced pro-fibrotic factors and enhanced epithelial healing.	[115]
AdV	IL-10	Corneal graft rejection	Topical	Sheep	In vivo	Substantially extended survival of corneal allografts and decreased rejection rates.	[134]
AdV	Flk-1	Corneal neovascularization	Anterior chamber	Rat	In vivo	Cauterized rats showed notable neovascularization inhibition.	[158]
Plasmid (shRNA)	MMP-9	Herpetic keratitis (HSV-1)	Corneal transfection	Mouse	In vivo	shRNA-mediated MMP-9 silencing inhibited corneal angiogenesis and reduced the severity of herpetic keratitis.	[244]
Electroporation	IL-10	Stromal keratitis	Plasmid electroporation	Mouse	In vivo	Gene expression under the CMV promoter remained robust for three days but declined by half each subsequent day. Switching from the CMV to the UbC promoter resulted in comparable durations of gene expression. Adding DNA nuclear-targeting sequences to vectors caused adverse effects.	[89]
Lipofection	KRT12	Meesmann’s epithelial corneal dystrophy (MECD)	Lipofectamine 2000	Human corneal limbal epithelial cells derived from limbal biopsies of patients with MECD	In vitro	Strong and targeted reduction in K12-Leu132Pro at both mRNA and protein levels.	[194]
Plasmid	OGFr (antisense cDNA)	Corneal epithelial wound healing	Gene gun	Rat	In vivo	Antisense OGFr gene transfer accelerated corneal re-epithelialization and increased epithelial DNA synthesis, whereas OGFr overexpression delayed wound healing.	[127]
CRISPR/dCas9	SOX2	Corneal endothelial wound healing	Corneal electroporation	Rat	In vivo	Activation of SOX2 resulted in decreased central corneal thickness and opacity relative to the control group. Overexpression of SOX2 led to elevated cell viability, proliferation rate, and S-phase cell count.	[88]
CRISPR-Cas9	TGFBI	Granular corneal dystrophy (GCD 2)	Transfection	Human, GCD 2 patient-derived corneal keratocytes	In vitro	Gene correction efficiency for the R124H mutation linked to GCD2 disorder was achieved without any off-target effects. In heterozygous cells, the correction rate was 20.6%, while in homozygous cells, it reached 41.3%.	[200]
Lipofection	TGFBIArg124Cys	Lattice corneal dystrophy type I (LCDI)	Lipofectamine 2000	Human corneal limbal epithelial cells derived from limbal biopsies of patients with LCD type I	Ex vivo	The siRNA targeting TGFBI-Arg124Cys effectively inhibited the expression of the mutated allele.	[199]
AdV/CRISPR-Cas9	COL8A2	Fuchs endothelial corneal dystrophy (FECD)	Intracameral	Mouse	In vivo	Efficient knockout of the mutant COL8A2 allele restored corneal endothelial pump function, maintained long-term corneal transparency, and showed no detectable adverse effects.	[206]
AAV9/CRISPR-Cas9	ICP0, ICP27	Herpetic keratitis	Intravenous injection	Rabbit	In vivo	Dual-target CRISPR/Cas9 significantly reduced HSV-1 viral shedding, eliminated viral shedding in 92% of treated eyes, and reduced latent HSV-1 expression in the trigeminal ganglia.	[191]
Dual AAV/CRISPR-Cas9	ICP4	Herpetic stromal keratitis	Retro-orbital injection	Mouse	In vivo	CRISPR-mediated ICP4 disruption combined with HSV-1-neutralizing antibody expression significantly reduced viral titers in the cornea, trigeminal ganglia, and brain.	[192]

## 8. Discussion

Gene therapy has emerged as a transformative therapeutic strategy with the potential to address both inherited and acquired corneal disorders by targeting the underlying molecular mechanisms rather than merely alleviating clinical manifestations. Over the past two decades, remarkable advances in vector engineering, gene-editing technologies, and ocular drug delivery have significantly expanded the therapeutic landscape of corneal gene therapy. Although clinical translation has progressed more rapidly in retinal diseases, culminating in the approval of the first ocular gene therapy for inherited retinal degeneration, the development of gene-based therapies for corneal diseases has remained largely at the preclinical stage despite encouraging experimental outcomes [245].

Current evidence demonstrates that gene therapy has shown therapeutic efficacy across a broad spectrum of corneal pathologies, including fibrosis, epithelial wound healing, corneal neovascularization, infectious keratitis, graft rejection, and inherited metabolic disorders. Most of these investigations have been performed in murine, rabbit, and ex vivo human corneal models, providing compelling proof-of-concept for disease-specific gene modulation [246,247]. Collectively, these studies indicate that therapeutic success depends not only on the selection of appropriate target genes but also on the optimization of delivery vectors and administration routes to achieve efficient and sustained transgene expression while minimizing toxicity.

Among the currently available delivery platforms, AAV vectors have emerged as the most extensively investigated and clinically promising system for corneal gene delivery because of their favorable safety profile, relatively low immunogenicity, and ability to mediate long-term gene expression in ocular tissues. Numerous studies have demonstrated the therapeutic efficacy of AAV-mediated delivery of antifibrotic and antiangiogenic genes, including Smad7, decorin, Id3, BMP7, HGF, and HLA-G, resulting in reduced corneal fibrosis, suppression of neovascularization, improved corneal transparency, and prolonged graft survival in preclinical models [73,118,120,238,247,248,249]. Nevertheless, adenoviral, lentiviral, and non-viral delivery systems continue to play important roles owing to their high transduction efficiency, larger transgene capacity, or improved biosafety characteristics, underscoring that each vector possesses distinct advantages and limitations and that the optimal delivery platform should be selected according to the target corneal layer, therapeutic gene size, disease characteristics, and desired duration of transgene expression.

One of the most significant developments in recent years has been the rapid evolution of genome-editing technologies, particularly CRISPR/Cas9 [41,250]. Compared with conventional gene supplementation strategies, CRISPR-based approaches provide the opportunity to permanently correct pathogenic mutations or precisely modulate disease-associated genes. Furthermore, the emergence of advanced editing platforms, including base editing and prime editing, has substantially improved editing precision while reducing unwanted genomic alterations, thereby expanding the therapeutic possibilities for inherited corneal disorders [41]. Although most CRISPR-based applications remain experimental, these technologies hold considerable promise for treating monogenic corneal dystrophies and other genetically determined corneal diseases that are difficult to manage using conventional pharmacological approaches. However, challenges including efficient delivery to target corneal cells, potential off-target genome editing, and the immunogenicity of CRISPR-associated proteins must be addressed before widespread clinical translation can be achieved [41]. Collectively, these advances are expected to facilitate precision medicine approaches by enabling mutation-specific therapeutic interventions tailored to individual patients.

Another notable trend is the increasing emphasis on regulating pathological signaling pathways involved in inflammation, fibrosis, angiogenesis, apoptosis, and immune responses rather than simply replacing defective genes. Therapeutic modulation of the TGF-β/Smad pathway, VEGF-mediated angiogenesis, inflammatory cytokines, and immune checkpoint molecules has consistently demonstrated beneficial effects in preventing corneal scarring, promoting epithelial regeneration, suppressing neovascularization, and improving graft survival [73,138,142,238,247,248,249]. Similarly, antiviral gene therapies targeting HSV have shown encouraging efficacy in reducing viral reactivation and limiting recurrent corneal infection, suggesting that gene therapy may provide durable therapeutic benefits for chronic infectious corneal diseases [251,252]. These findings highlight the versatility of gene therapy as a platform capable of targeting multiple pathogenic pathways involved in both inherited and acquired corneal disorders.

Despite these encouraging advances, the translation of corneal gene therapy into routine clinical practice remains limited. Most published studies have been conducted in small-animal models with relatively short follow-up periods, and only a limited number have evaluated long-term safety, sustained transgene expression, or therapeutic durability [253,254,255]. Furthermore, although several gene therapy clinical trials have been initiated for systemic manifestations of mucopolysaccharidoses, their effects on corneal opacity and visual outcomes remain insufficiently understood [256]. Likewise, despite the growing understanding of the genetic basis of inherited corneal dystrophies, relatively few investigations have translated these discoveries into targeted gene therapies suitable for clinical application.

Overall, the accumulated evidence indicates that corneal gene therapy is transitioning from proof-of-concept experimentation toward disease-specific therapeutic development. Continued improvements in vector engineering, genome-editing technologies, tissue-specific promoters, and targeted ocular delivery systems are expected to accelerate clinical translation. Future therapeutic strategies will likely integrate precise genome editing with advanced biomaterials, nanotechnology-based delivery systems, stem cell therapy, and regenerative medicine approaches, thereby enabling safer, more durable, and personalized treatments for a wide range of corneal diseases [257]. Collectively, these multidisciplinary advances are expected to establish gene therapy as an integral component of precision ophthalmology, providing safe, targeted, and long-lasting therapeutic options for both inherited and acquired corneal diseases [258,259].

## 9. Challenges and Future Perspectives

Corneal transplantation remains the gold standard for the treatment of corneal blindness. However, the persistent shortage of donor corneas, graft rejection, limited graft survival, and challenges associated with donor tissue procurement, preservation, and storage have accelerated the search for alternative therapeutic strategies [260]. Gene therapy has emerged as a promising approach because it addresses the underlying molecular mechanisms of disease rather than simply replacing damaged tissue, thereby offering the potential to restore corneal function, preserve vision, and prevent disease progression. Encouraging preclinical studies have demonstrated therapeutic benefits in epithelial wound healing, corneal fibrosis, neovascularization, graft survival, herpetic keratitis, keratoconus, LSCD, FECD, and several inherited corneal dystrophies [73,110,131,195,261,262]. Nevertheless, despite these promising findings, the successful translation of corneal gene therapy from experimental models to routine clinical practice remains hindered by multiple biological, technical, immunological, and regulatory challenges (Figure 9).

One of the major challenges is the development of efficient, safe, and disease-specific gene delivery systems capable of achieving sustained therapeutic gene expression while minimizing adverse effects. The rAAV vectors remain the most widely investigated delivery platform because of their favorable safety profile, low pathogenicity, and ability to mediate long-term transgene expression in ocular tissues. However, their limited packaging capacity (~4.7 kb) restricts the delivery of large therapeutic genes and advanced genome-editing platforms, including base editors and prime editors [67,97]. Consequently, novel packaging strategies, including dual-AAV vectors, split-intein protein reconstitution, trans-splicing AAV vectors, and mini-gene constructs, are being developed to overcome these limitations and expand the therapeutic applicability of AAV-mediated corneal gene therapy [108]. Furthermore, vector optimization remains essential because transduction efficiency and the magnitude and duration of transgene expression vary considerably among AAV serotypes, delivery routes, and corneal cell types, necessitating careful vector selection and dose optimization to achieve therapeutic efficacy while minimizing toxicity [71].

The immunogenicity of viral vectors represents another major obstacle to clinical translation. Although the cornea possesses relative immune privilege, pre-existing or treatment-induced neutralizing antibodies against AAV capsid proteins may significantly reduce vector transduction efficiency, compromise long-term transgene expression, and limit repeated vector administration. In addition, immune responses against viral capsids may increase ocular inflammation and reduce therapeutic efficacy, particularly in inflamed or diseased corneas [263,264].

Despite the advantages of viral vectors, currently available non-viral delivery systems also face important limitations. Although LPs, polymeric nanoparticles, extracellular vesicles, hydrogels, biomaterial-based carriers, and transposon systems provide larger cargo capacity and lower immunogenicity, their transfection efficiency, tissue penetration, long-term stability, and sustained therapeutic expression in corneal tissues remain inferior to those achieved with viral vectors. These limitations continue to restrict their widespread clinical application [265,266].

Beyond vector-associated challenges, the immunogenicity and safety of genome-editing cargo remain important concerns. CRISPR-associated nucleases, including Cas9, originate from bacterial species and may elicit both humoral and cellular immune responses, potentially reducing editing efficiency and inducing inflammatory reactions. Moreover, prolonged nuclease expression increases the risk of off-target genome editing, unintended genomic alterations, chromosomal rearrangements, and persistent immune activation, raising concerns regarding long-term genomic stability and patient safety [42].

Ethical considerations surrounding genome editing in ocular tissues also warrant attention. Although corneal gene therapy is generally directed toward somatic cells and is therefore not intended to modify the germline, the potential for off-target alterations, unintended genomic changes, and uncertain long-term consequences requires careful consideration [41,267,268]. Appropriate informed consent should clearly communicate the potential benefits, uncertainties, and long-term risks associated with genome-editing interventions. In addition, rigorous preclinical safety assessment, long-term monitoring, transparent reporting of adverse events, and equitable access to potentially costly therapies will be essential for responsible clinical translation [267,269].

Another major challenge is achieving efficient, tissue-specific delivery and precisely regulated transgene expression within the cornea [270]. Therapeutic targets differ considerably among corneal diseases; for example, limbal stem cell deficiency requires selective targeting of limbal epithelial stem cells, keratoconus and stromal fibrosis primarily require stromal keratocyte targeting, whereas FECD depends on efficient gene delivery to corneal endothelial cells [110]. Furthermore, anatomical barriers including the tear film, blinking, epithelial tight junctions, rapid tear turnover, and the dense stromal ECM substantially reduce vector retention and transduction efficiency, making efficient gene delivery particularly challenging.

Corneal wound-healing dynamics may further influence the efficacy of gene therapy, particularly in inflamed or vascularized corneas. Following injury, epithelial disruption, stromal remodeling, inflammatory-cell infiltration, and neovascularization can substantially alter the local tissue microenvironment and affect vector penetration, cellular uptake, and transgene expression. Persistent inflammation and vascularization may also compromise corneal immune privilege and increase the risk of immune-mediated responses to therapeutic vectors. Therefore, the timing of gene delivery relative to the stage of wound healing and the inflammatory status of the cornea should be carefully considered when designing therapeutic strategies and future clinical protocols [271,272,273].

The durability of therapeutic gene expression is another important consideration because corneal tissues exhibit different rates of cellular turnover and regenerative capacity. In particular, the corneal epithelium undergoes continuous renewal, with the epithelial layer undergoing approximately complete turnover within 1–2 weeks, which may result in dilution or loss of therapeutic expression when transduced cells are replaced [274,275]. In contrast, relatively quiescent stromal keratocytes may permit more sustained expression, although injury or disease can induce keratocyte activation, differentiation, or cell loss, potentially affecting long-term transgene persistence. Corneal endothelial cells are largely postmitotic in vivo and may therefore support more sustained expression, although their limited proliferative capacity requires careful consideration of long-term safety [276]. Accordingly, vector persistence, promoter activity, target-cell turnover, and the disease-specific regenerative environment should be evaluated when determining the expected duration of therapeutic effects and the potential need for repeat administration.

Genome-editing safety remains another critical consideration before widespread clinical implementation. Although CRISPR/Cas technology has revolutionized precision medicine, unintended off-target mutations, chromosomal rearrangements, and prolonged nuclease activity remain important concerns, particularly in transparent tissues such as the cornea, where even minor structural alterations may compromise vision [41,277]. These risks are especially relevant for inherited disorders such as TGFBI-associated corneal dystrophies and FECD, where highly precise correction of pathogenic mutations is essential to restore normal corneal structure and function.

Clinical translation is further complicated by the considerable genetic heterogeneity of corneal diseases. The identification and validation of disease-causing genes and patient-specific mutations remain costly and time-consuming, particularly for genetically diverse disorders such as corneal dystrophies and keratoconus [110]. Furthermore, gene therapy is generally most effective during the early stages of disease progression, when sufficient healthy corneal cells remain capable of expressing the therapeutic gene. In this context, gene therapy should be considered primarily as a disease-modifying and potentially transplantation-sparing strategy rather than a universal replacement for corneal transplantation. In early or moderate disease, gene therapy may slow disease progression, preserve viable corneal tissue, and potentially delay the need for transplantation [278,279]. However, in advanced disease characterized by extensive stromal scarring, limbal stem cell depletion, or severe endothelial cell loss, gene therapy alone may be insufficient to restore corneal transparency and function, requiring complementary approaches such as stem cell therapy, tissue engineering, or corneal transplantation. Gene therapy may also have a complementary role in transplantation by modifying disease-associated pathways or the ocular microenvironment and potentially improving graft survival or reducing graft rejection [240,279,280]. Thus, future clinical strategies may adopt a disease-stage-dependent approach in which gene therapy is used to preserve corneal function and postpone transplantation when feasible, while combination approaches may be considered for advanced disease [270]. In addition, currently available animal models do not completely replicate the anatomy, biomechanics, immune responses, and disease progression observed in human corneas, limiting the predictive value of preclinical studies [281].

Finally, several practical barriers continue to impede the clinical implementation of corneal gene therapy. To date, no gene therapy has received regulatory approval (e.g., FDA or EMA) for the treatment of corneal diseases, reflecting the substantial gap between encouraging preclinical findings and successful clinical translation. Large-scale vector production, Good Manufacturing Practice (GMP)-compliant manufacturing, quality control, long-term safety monitoring, regulatory approval, and commercialization remain technically demanding and economically expensive [282]. From a translational perspective, viral and nonviral delivery systems also differ in their manufacturability and scalability. Viral vectors, particularly AAV-based systems, require complex production, purification, characterization, and quality-control processes to ensure consistent potency, purity, and safety, which may increase manufacturing complexity and cost [283,284]. In contrast, nonviral systems, including lipid nanoparticles and polymeric nanoparticles, generally offer greater flexibility for large-scale formulation and cargo modification. However, maintaining consistent particle characteristics, cargo loading, stability, biological activity, and tissue-specific delivery remains challenging for nonviral platforms [109,285]. Therefore, the selection of viral or nonviral delivery systems should consider not only transduction efficiency and durability but also manufacturing feasibility, scalability, reproducibility, cost, and regulatory requirements. Moreover, the high costs associated with vector development, genome sequencing, mutation identification, individualized therapeutic design, and long-term patient follow-up continue to limit the accessibility and widespread adoption of gene therapy for corneal diseases [286].

Future research should focus on developing next-generation gene delivery platforms with improved safety, tissue specificity, and long-term therapeutic efficacy (Figure 9). Advances in novel AAV packaging strategies, including dual-AAV vectors, split-intein technologies, trans-splicing AAV systems, engineered mini-gene constructs, and next-generation AAV capsid engineering through rational design, directed evolution, and artificial intelligence (AI)-assisted AAV capsid engineering, are expected to overcome current limitations in cargo capacity, transduction efficiency, tissue specificity, immune evasion, and immunogenicity [264]. Furthermore, the integration of high-fidelity CRISPR systems, base editing, prime editing, epigenome editing, and RNA editing may substantially improve the precision and safety of gene correction while minimizing off-target effects [42]. In addition, transient immunomodulation, antibody depletion strategies, and the development of immune-evasive vectors may facilitate repeated vector administration and improve the long-term durability of gene therapy [263]. The development of cornea- and cell-specific promoters, inducible gene expression systems, and cell-targeted delivery strategies will further enhance targeted gene expression in corneal epithelial cells, stromal keratocytes, limbal epithelial stem cells, and corneal endothelial cells [270].

Future studies should also investigate combination therapeutic strategies integrating gene therapy with stem cells, extracellular vesicles, biomaterials, anti-fibrotic agents, anti-angiogenic therapies, and immunomodulatory approaches to simultaneously target multiple pathogenic pathways involved in corneal diseases and promote functional corneal regeneration. For example, combining antifibrotic gene modulation with therapeutic approaches that inhibit corneal neovascularization, suppress inflammation, or enhance epithelial regeneration may provide greater therapeutic benefits than single-gene interventions [287].

In addition, precision medicine approaches based on comprehensive genetic profiling, patient-specific mutation analysis, and early molecular diagnosis should be emphasized to enable individualized therapeutic interventions. Emerging technologies, including guide RNA optimization, patient-derived induced pluripotent stem cell (iPSC)-derived corneal organoids, organ-on-chip platforms, and advanced preclinical disease models, are expected to accelerate vector development, improve therapeutic outcome prediction, and provide clinically relevant platforms for preclinical evaluation [288,289].

Future clinical trials will also require carefully defined patient-selection criteria to maximize therapeutic benefit and ensure safety. Relevant considerations may include confirmed genetic diagnosis and mutation type for inherited disorders, disease stage and severity, the extent of corneal scarring or neovascularization, residual viable target cells, ocular surface and inflammatory status, and previous surgical or medical interventions [278,279,290]. Early-stage disease with sufficient viable target cells may be more amenable to gene-based intervention, whereas advanced disease with extensive fibrosis, limbal stem cell deficiency, or severe endothelial cell loss may require combination approaches. Patient stratification based on molecular and clinical characteristics will therefore be important for evaluating therapeutic efficacy and identifying individuals most likely to benefit from gene therapy [278,279]. Finally, overcoming challenges associated with large-scale GMP-compliant manufacturing, standardized quality control, regulatory approval, treatment cost, and long-term safety evaluation through well-designed multicenter clinical trials will be essential for translating promising experimental findings into routine clinical practice [291].

Collectively, these multidisciplinary advances are expected to transform corneal gene therapy from a predominantly preclinical approach into a clinically applicable, safe, durable, and personalized therapeutic modality for inherited and acquired corneal diseases.

## 10. Conclusions

Gene therapy has emerged as a promising therapeutic approach for the treatment of inherited and acquired corneal diseases by targeting their underlying molecular mechanisms. Advances in gene-editing technologies and gene delivery systems have demonstrated encouraging therapeutic outcomes in preclinical studies. Although challenges related to vector safety, delivery efficiency, long-term transgene expression, and clinical translation remain, ongoing improvements in these areas are expected to accelerate the development of safe and effective gene-based therapies, ultimately reducing reliance on corneal transplantation and improving visual outcomes. Continued advances in vector engineering, precision genome-editing technologies, tissue-specific gene delivery, and combination therapeutic strategies are expected to further facilitate the clinical translation of corneal gene therapy. Collectively, these innovations have the potential to establish gene therapy as a safe, targeted, and durable therapeutic modality, advancing precision ophthalmology and improving the management of a broad spectrum of inherited and acquired corneal diseases.

## Figures and Tables

**Figure 1:**
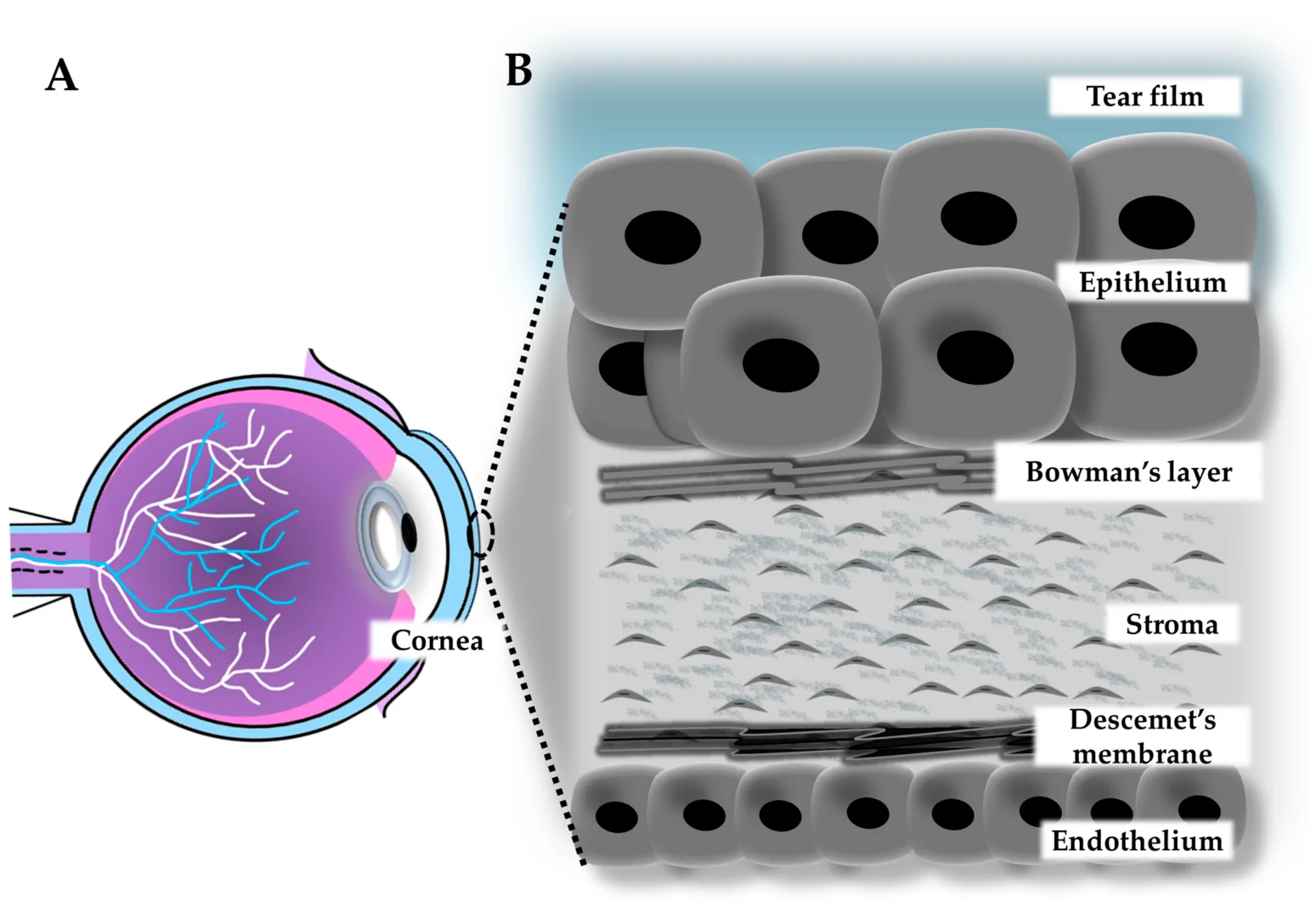
Schematic illustration of the anatomical structure of the cornea. (**A**) The anatomical location of the cornea within the eye. (**B**) Enlarged schematic showing the layered organization of the cornea, including the tear film, corneal epithelium, Bowman’s layer, stroma, Descemet’s membrane, and corneal endothelium.

**Figure 2:**
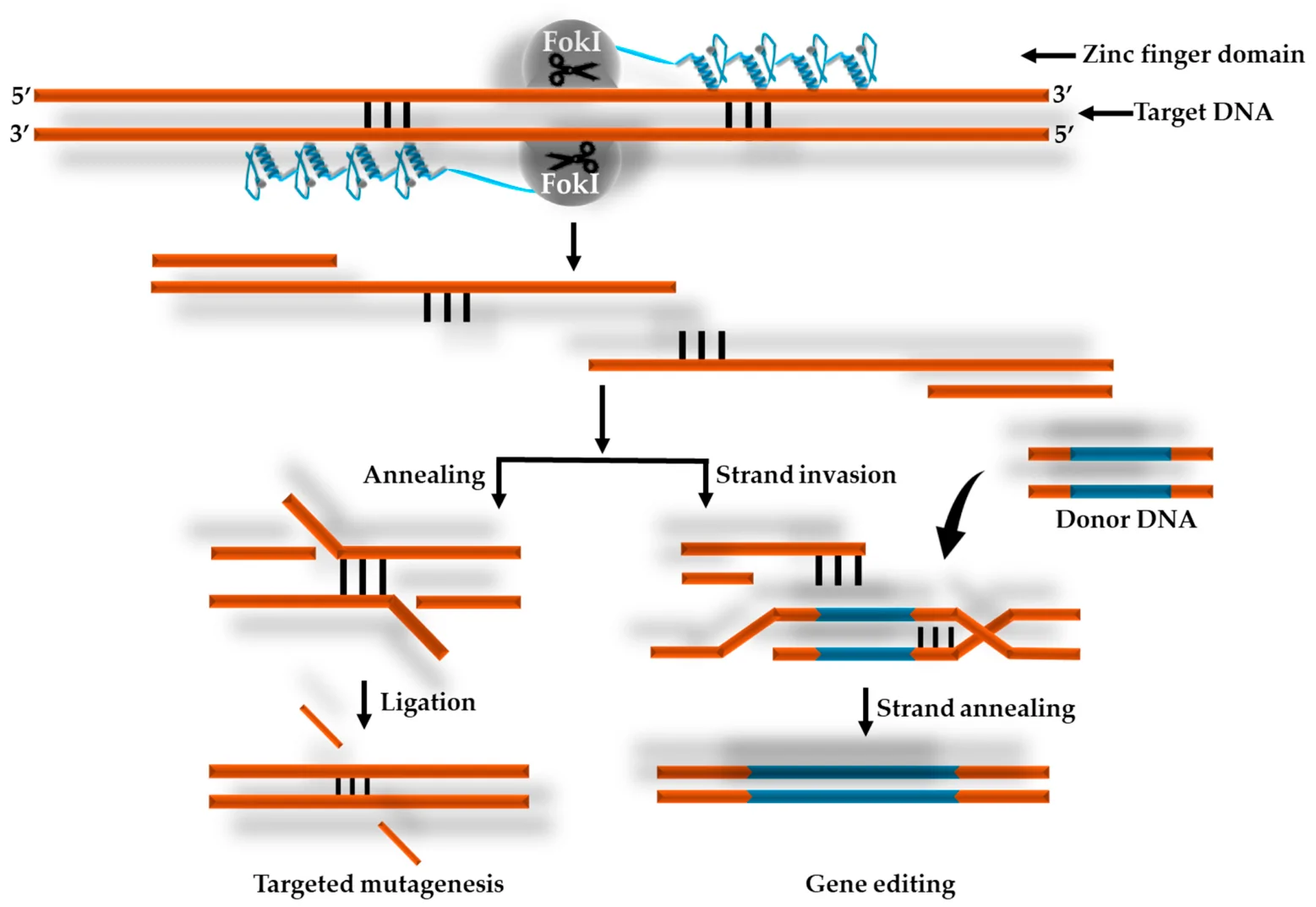
Mechanism of ZFN-mediated genome editing. Two ZFN monomers bind adjacent target DNA sequences through their zinc finger DNA-binding domains, enabling dimerization of the FokI nuclease domains and the generation of a site-specific DNA double-strand break. The resulting DNA damage is repaired either by non-homologous end joining (NHEJ), leading to targeted mutagenesis, or by homology-directed repair (HDR) in the presence of a donor DNA template, enabling precise gene correction, insertion, or replacement.

**Figure 3:**
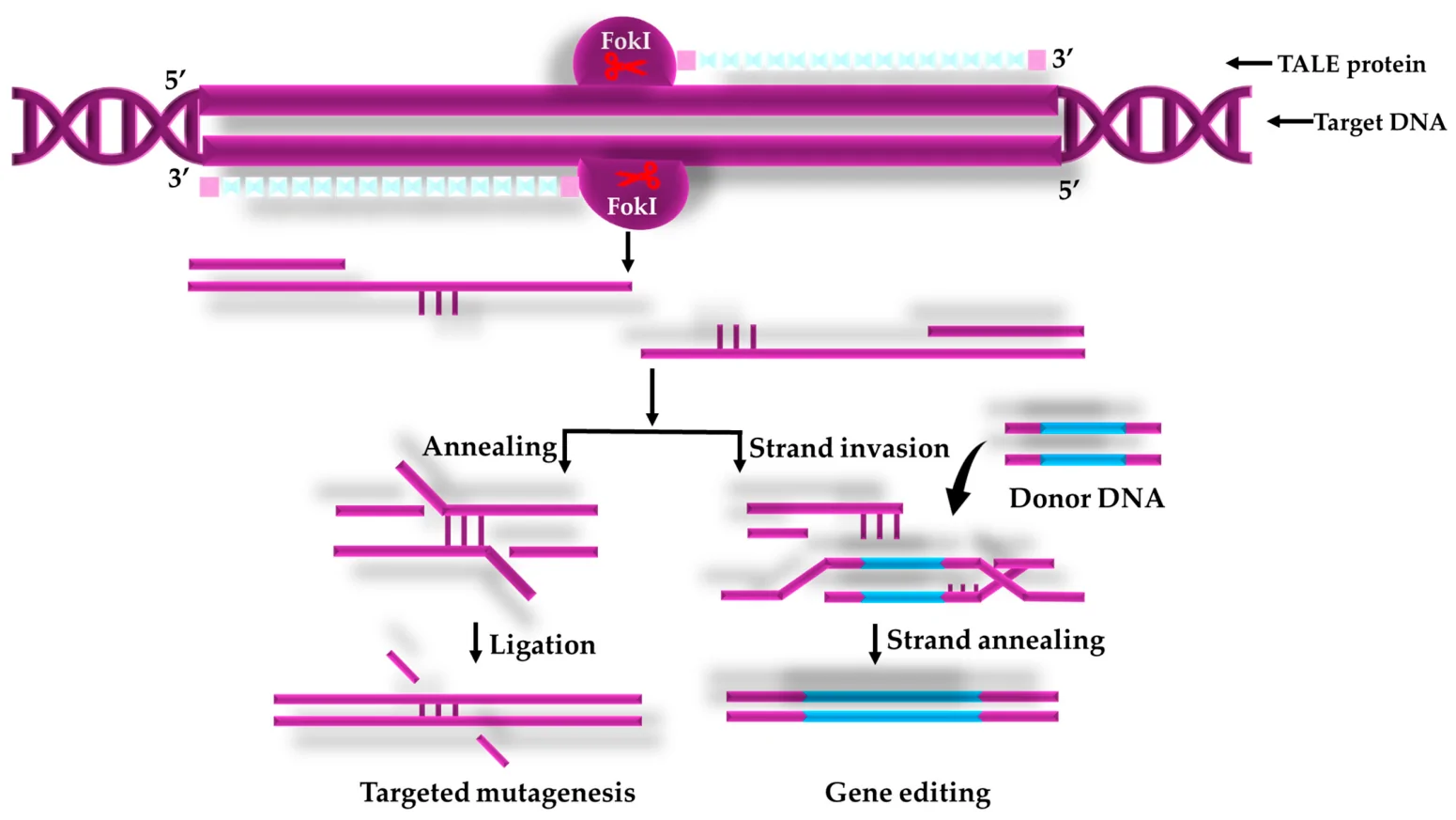
Mechanism of transcription activator-like effector nuclease-mediated genome editing. Engineered transcription activator-like effector DNA-binding domains recognize specific target DNA sequences, enabling dimerization of the FokI endonuclease and generation of a site-specific DNA double-strand break. The resulting DNA damage is repaired through non-homologous end joining, leading to targeted mutagenesis, or through homology-directed repair (HDR) in the presence of a donor DNA template, enabling precise gene correction, insertion, or replacement.

**Figure 4:**
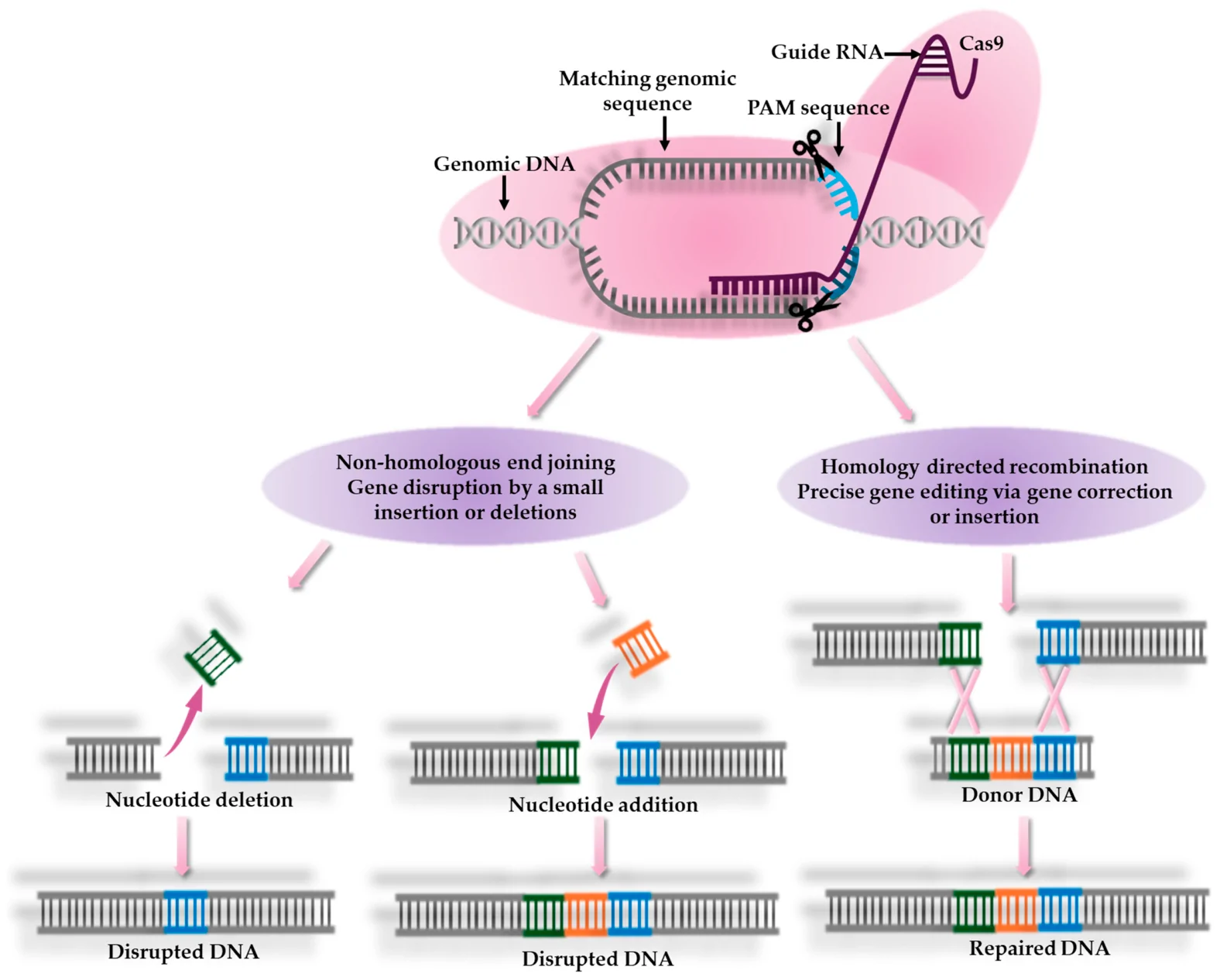
Mechanism of clustered regularly interspaced short palindromic repeats (CRISPR)/CRISPR-associated protein 9 (Cas9)-mediated genome editing. The guide RNA directs the Cas9 endonuclease to a complementary target DNA sequence adjacent to a protospacer adjacent motif (PAM), where Cas9 generates a site-specific DNA double-strand break. The resulting DNA damage is repaired either through non-homologous end joining (NHEJ), leading to insertion or deletion mutations that disrupt gene function, or through homology-directed repair (HDR) in the presence of a donor DNA template, enabling precise gene correction, insertion, or replacement.

**Figure 5:**
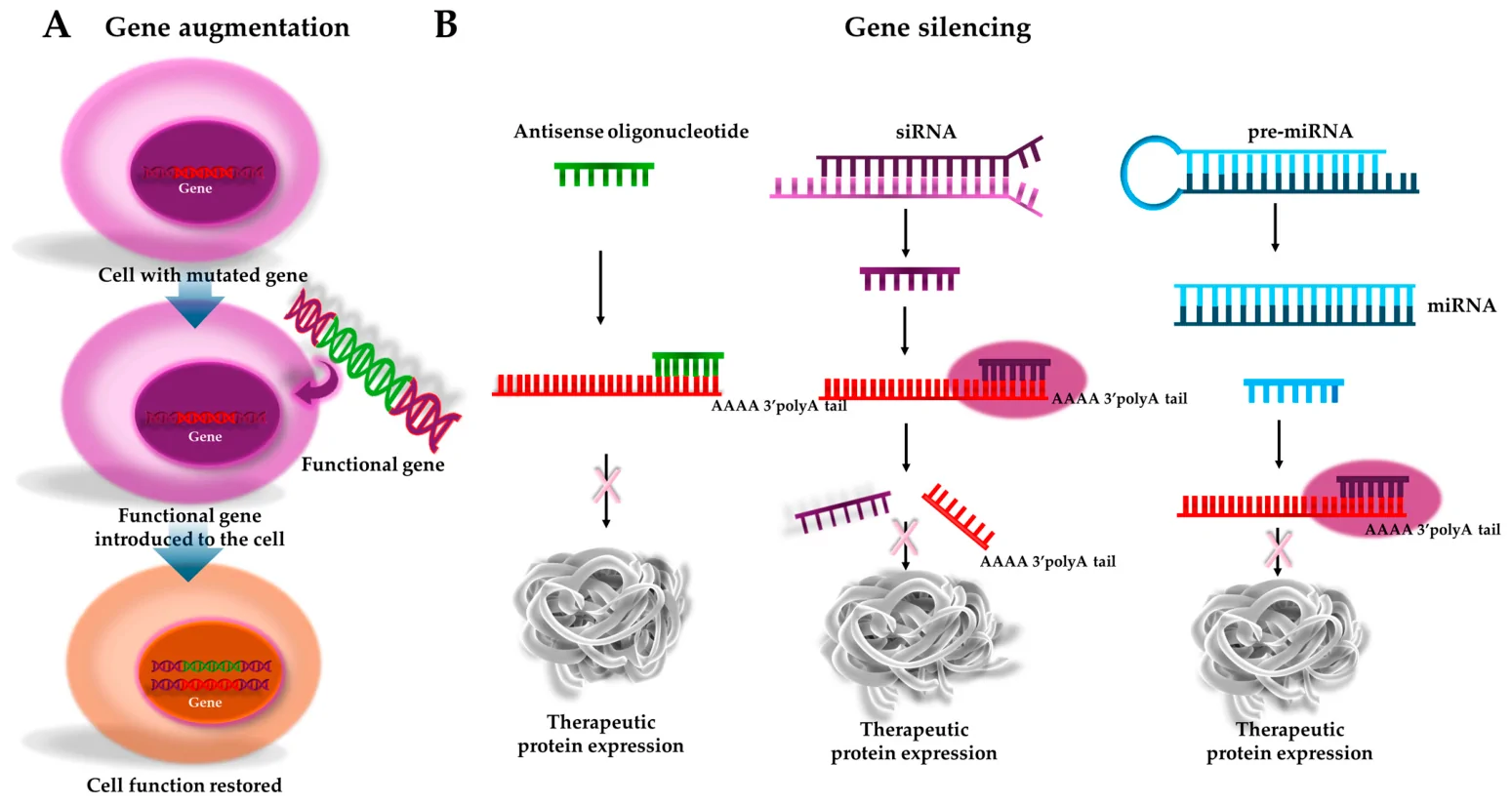
Major gene therapy strategies used for corneal diseases. (**A**) Schematic illustration of gene augmentation, in which a functional therapeutic gene is delivered into target cells to restore or enhance protein expression. (**B**) Schematic representation of gene silencing approaches, including small interfering RNA (siRNA), short hairpin RNA (shRNA), microRNA (miRNA), and antisense oligonucleotides (ASOs), which suppress disease-associated gene expression through mRNA degradation, translational repression, or modulation of pre-mRNA splicing. These complementary strategies enable targeted regulation of pathogenic genes and represent important therapeutic approaches for inherited and acquired corneal diseases.

**Figure 6:**
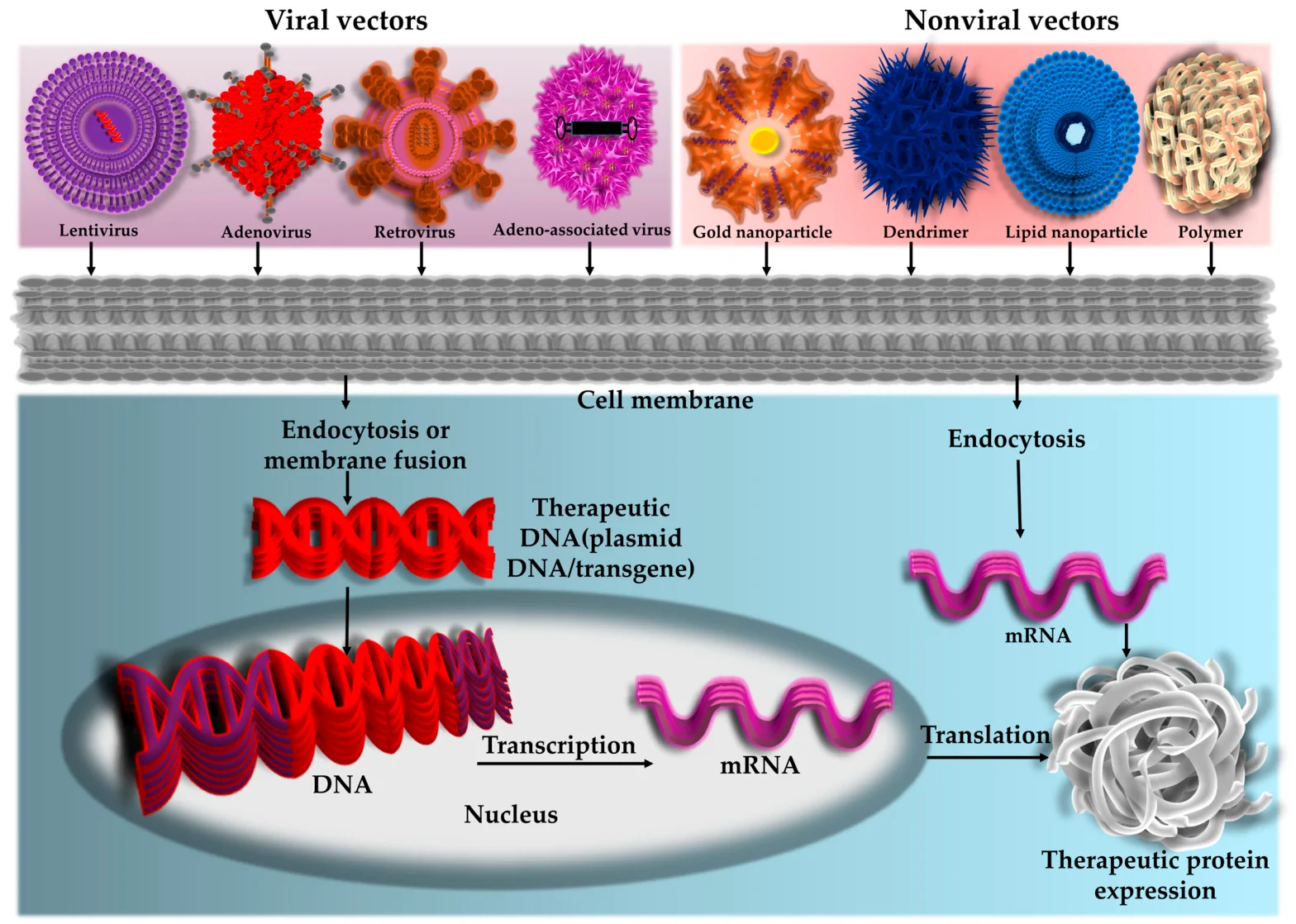
Schematic illustration of the mechanisms of viral and nonviral gene delivery. Viral vectors (lentiviral vectors, adenoviral vectors, retroviral vectors, and adeno-associated viral vectors) and nonviral vectors (gold nanoparticles, dendrimers, lipid nanoparticles, and polymers) facilitate intracellular delivery of therapeutic nucleic acids through distinct cellular uptake mechanisms. Following cellular internalization, the delivered DNA or RNA undergoes intracellular processing, transcription, and translation, resulting in therapeutic protein expression.

**Figure 7:**
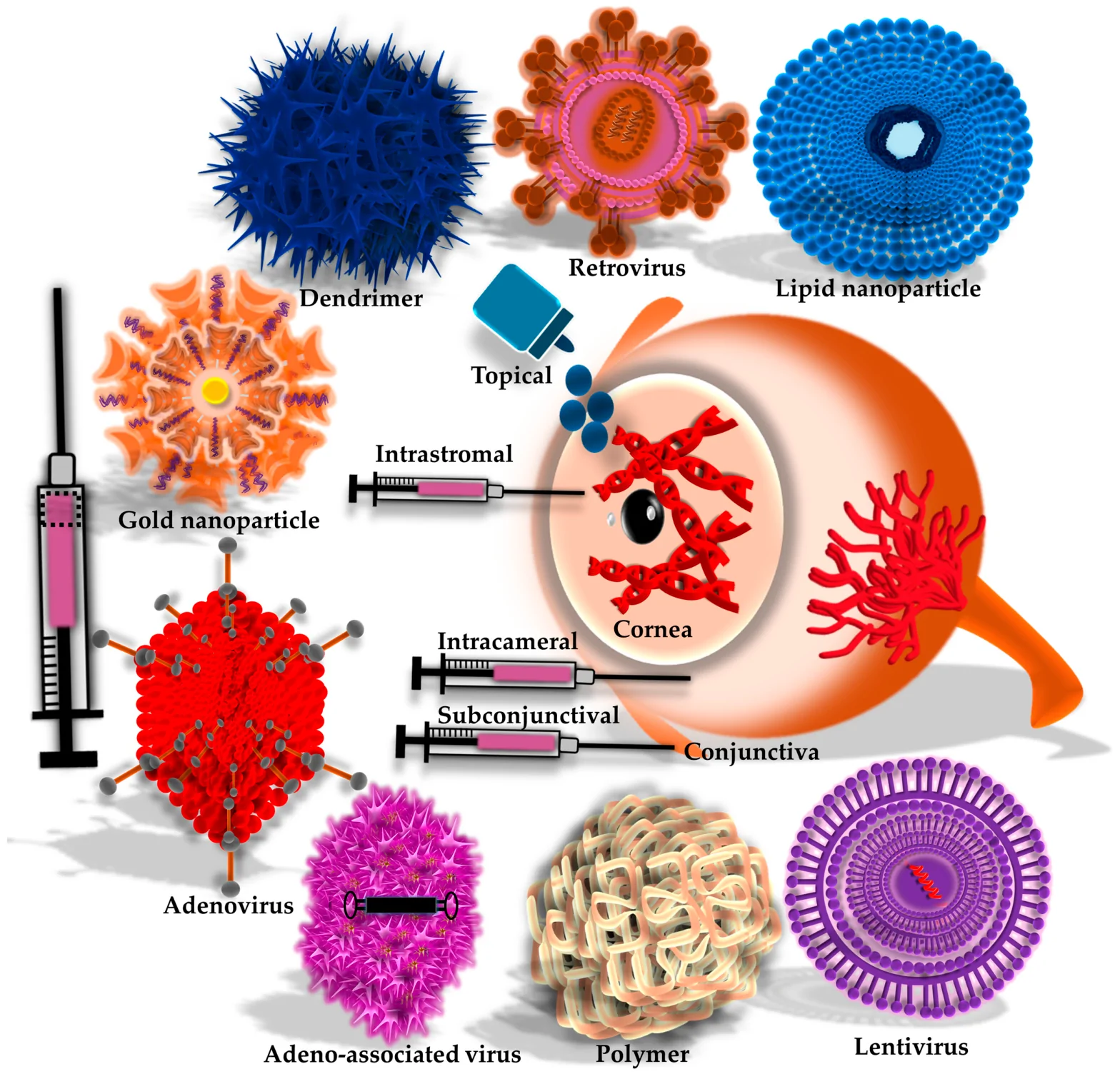
Schematic illustration of viral and nonviral vectors and their routes of administration for corneal gene therapy. Viral vectors, including adenoviral vectors, adeno-associated viral vectors, lentiviral vectors, and retroviral vectors, and nonviral vectors, including lipid nanoparticles, polymers, dendrimers, and gold nanoparticles, are commonly employed for therapeutic gene delivery to the cornea. Depending on the target corneal layer and therapeutic objective, gene delivery can be achieved through topical administration, subconjunctival injection, intrastromal injection, intracameral injection, intravitreal injection, or systemic administration. The choice of vector and delivery route influences transduction efficiency, tissue specificity, therapeutic efficacy, and safety.

**Figure 8:**
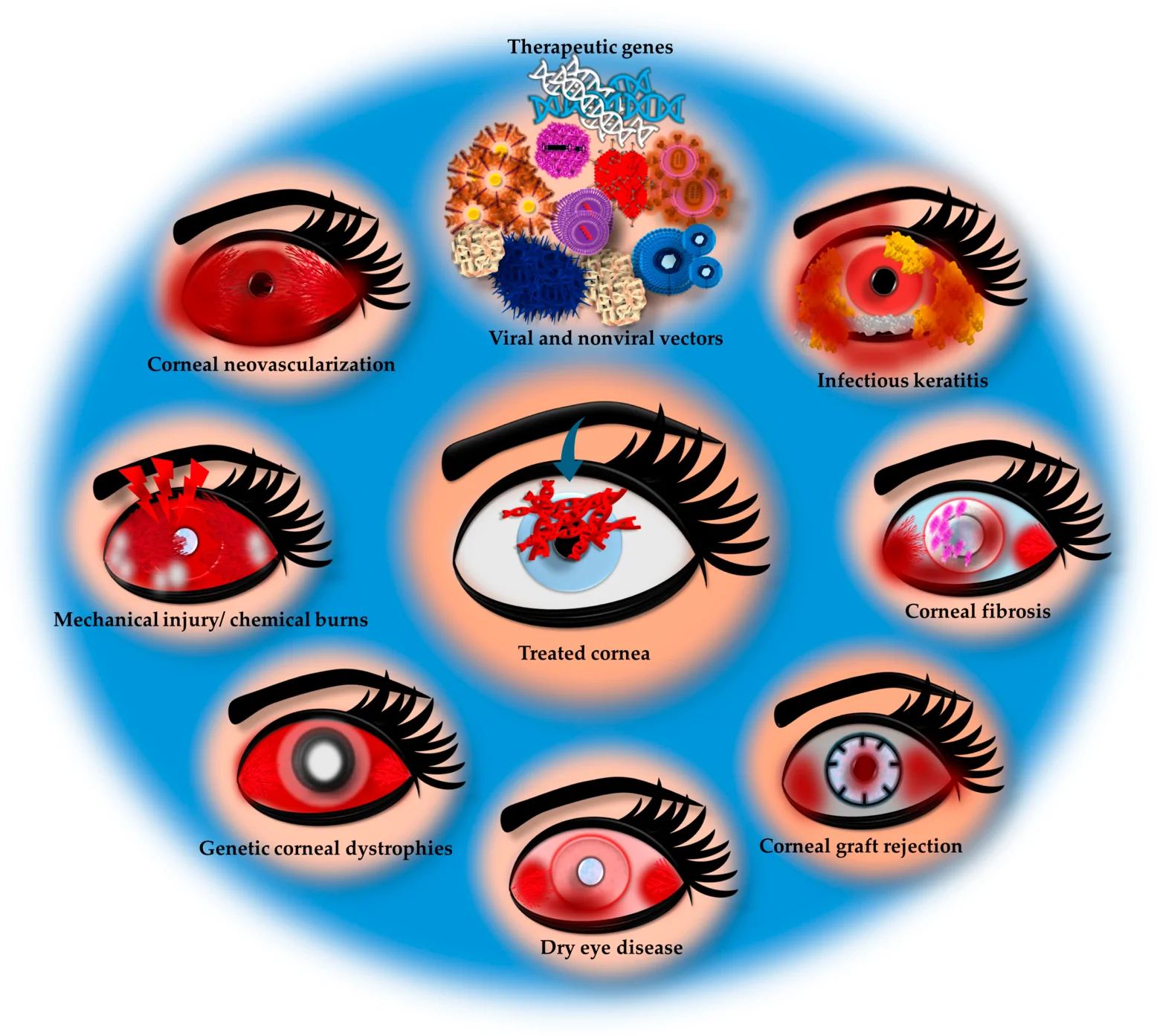
Therapeutic applications of gene therapy in corneal diseases. Schematic illustration of the major corneal diseases currently investigated for gene therapy. The figure highlights representative therapeutic targets for corneal neovascularization, infectious keratitis, mechanical injuries and chemical burns, corneal fibrosis, corneal graft rejection, dry eye disease, and genetic corneal dystrophies. Gene therapy employs therapeutic genes and gene-editing molecules delivered through viral and nonviral vectors to modulate disease-associated molecular pathways, suppress inflammation, fibrosis, neovascularization, and infection, promote corneal repair and regeneration, and ultimately restore corneal transparency and visual function.

**Figure 9:**
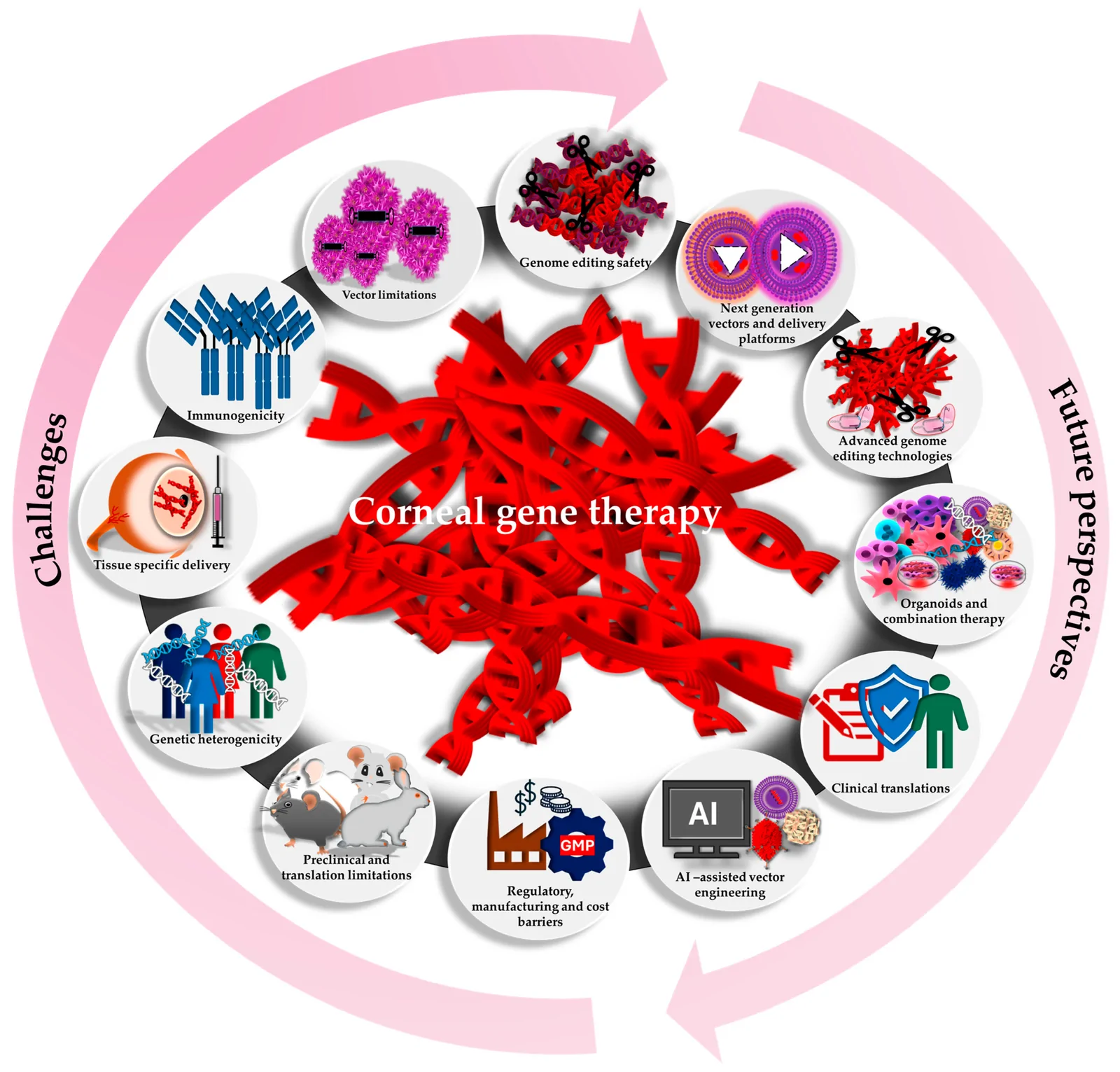
Challenges and future perspectives of corneal gene therapy. Major challenges limiting the clinical translation of corneal gene therapy include vector-related limitations, tissue-specific gene delivery, immunogenicity, genome-editing safety, genetic heterogeneity, preclinical and translational limitations, and regulatory, manufacturing, and cost barriers. Future advances are expected to focus on next-generation viral and nonviral vectors, advanced genome-editing technologies, artificial intelligence-assisted vector engineering, organoid- and combination therapy-based approaches, and strategies to facilitate safe and effective clinical translation.

## Data Availability

Data is contained within the article. The original contributions presented in this study are included in the article. Further inquiries can be directed to the corresponding author.

## Abbreviations

AAV	Adeno-associated virus
AdV	Adenovirus
AI	Artificial Intelligence
Cas9	CRISPR-associated protein 9
CRISPR	Clustered Regularly Interspaced Short Palindromic Repeats
DALK	Deep Anterior Lamellar Keratoplasty
DED	Dry Eye Disease
DMEK	Descemet Membrane Endothelial Keratoplasty
DSEK	Descemet Stripping Endothelial Keratoplasty
ECM	Extracellular Matrix
FECD	Fuchs Endothelial Corneal Dystrophy
GMP	Good Manufacturing Practice
HDR	Homology-Directed Repair
HLA-G	Human Leukocyte Antigen-G
HSV	Herpes Simplex Virus
IFN	Interferon
JECD	Juvenile Epithelial Corneal Dystrophy
LCD	Lattice Corneal Dystrophy
LNPs	Lipid Nanoparticles
LV	Lentivirus
miRNA	MicroRNA
ZFNs	Zinc Finger Nucleases
TGF-β	Transforming Growth Factor Beta
TALE	Transcription Activator-Like Effector
TALENs	Transcription Activator-Like Effector Nucleases
PRK	Photorefractive Keratectomy
NHEJ	Non-Homologous End Joining
